# Aminoquinoline-Based Tridentate (*NNN*)-Copper Catalyst for C–N Bond-Forming Reactions from Aniline and Diazo Compounds

**DOI:** 10.3390/molecules29030730

**Published:** 2024-02-05

**Authors:** Mohsen Teimouri, Selvam Raju, Edward Acheampong, Allison N. Schmittou, Bruno Donnadieu, David O. Wipf, Brad S. Pierce, Sean L. Stokes, Joseph P. Emerson

**Affiliations:** 1 Department of Chemistry, Mississippi State University, Starkville, MS 39762, USAbdonnadieu@chemistry.msstate.edu (B.D.); dwipf@chemistry.msstate.edu (D.O.W.);; 2 Department of Chemistry and Biochemistry, The University of Alabama, 3097D Shelby Hall, Tuscaloosa, AL 35487, USA

**Keywords:** copper carbene, 8-aminoquinoline-based ligand, α-diazoesters, N–H insertion

## Abstract

A new tridentate Cu^2+^ complex based on (*E*)-1-(pyridin-2-yl)-*N*-(quinolin-8-yl)methanimine (PQM) was generated and characterized to support the activation of diazo compounds for the formation of new C–N bonds. This neutral Schiff base ligand was structurally characterized to coordinate with copper(II) in an equatorial fashion, yielding a distorted octahedral complex. Upon characterization, this copper(II) complex was used to catalyze an efficient and cost-effective protocol for C–N bond formation between *N*-nucleophiles and copper carbene complexes arising from the activation of diazo carbonyl compounds. A substrate scope of approximately 15 different amine-based substrates was screened, yielding 2° or 3° amine products with acceptable to good yields under mild reaction conditions. Reactivity towards phenol and thiophenol were also screened, showing relatively weak C–O or C–S bond formation under optimized conditions.

## 1. Introduction

Copper(II) complexes featuring tridentate nitrogen ligands exhibit remarkable versatility in their coordination numbers and geometries due to their d^9^ electronic configuration [1,2]. This diversity in coordination capability has paved the way for the creation of intricate structures with potential applications spanning material science, supramolecular architectures with captivating photoluminescent properties, as well as potential roles in anticancer and NSAID activity [3,4,5,6,7]. Furthermore, copper(II) complexes with planar nitrogen ligands have proven highly effective in the realm of bioinorganic chemistry, serving as reactive models for the active sites in the proteins involved in oxygen activation. The organic motifs, framework, and donor atom set of these complexes all wield pivotal roles in their chemistry [8,9]. These coordination complexes have also found extensive utility in catalysis, with numerous research groups delving into their catalytic properties across a wide spectrum of processes, including oxidation reactions [10], atom transfer reactions [11,12], Chan-Evans-Lam reactions [13,14,15,16], Ullmann and Goldberg-type couplings [17], and click reactions [18,19]. 

The focused exploration of carbon–nitrogen bond formation and cleavage has been a dynamic research area for several decades [20,21,22]. Dating back to 1901, various controlled methods for introducing amino groups have been pioneered by Ullmann, Goldberg, Chan, Evans, Lam, and Buchwald, with these advancements driven by the development and application of innovative ligands [23,24]. Many copper-catalyzed reactions entail the addition of heteroatom nucleophiles to α-diazo carbonyl compounds, yielding enolate intermediates [25,26]. α-Diazoesters and carbonyl compounds have gained broad utility in both pharmaceutical and laboratory settings for the synthesis of α-amino acid derivatives due to their tunable reactivity, encompassing electron acceptor and donor groups [27,28,29]. Despite significant strides in carbene transfer reactions in organic solvents, recent studies have emphasized the solvent’s influence on C–H, Si–H, O–H, and N–H activation in chemoselective insertion reactions [30,31]. Notably, in 2007, Hansen et al. reported the formation of cyclopropanes employing an expensive dimeric Rh_2_(OAc)_4_ catalyst with *N*-vinylphthalimide and methyl phenyldiazoacetate under reflux conditions, achieving clean reactions and high yields [32]. Recently, Lucie et al. reported electron-rich ruthenium phthalocyanines as a catalyst for the conversion of aromatic, heterocyclic, and aliphatic amines to corresponding substituted glycine derivatives [33]. Kantam and coworkers demonstrated that with nanocrystalline copper(II) oxide or Cu(acac)_2_ in ionic liquids, glycine esters could be prepared in good yields through the insertion of α-diazoacetate into the N–H bonds of amines [34,35].

Numerous endeavors have been dedicated to designing asymmetric carbene insertions into N–H bonds, and copper(II)-based complexes featuring a planar tridentate system have exhibited exceptional promise due to their robust ligand affinity [30,31,36,37,38,39]. Lee and coworkers demonstrated the conversion of α-aryl-α-diazo esters into glycine derivatives using a Cu(I)/planar-chiral bipyridine complex, providing critical insights into the development of copper/planar-chiral bipyridine for carbene-mediated transformations with high enantiomeric excess [29]. While achieving superior enantioselectivity, this protocol has limitations in substrate scope. Fasan and coworkers also detailed a biocatalytic approach for the enantioselective synthesis of α-trifluoromethyl amines via N–H bond insertion utilizing acceptor-acceptor carbene donors [40]. Sivasankar’s group reported the chemoselective utilization of copper(I) complexed with an air-stable phosphine ligand for N–H insertion over O–H [41]. Transition metals have earned acclaim for their efficacy in catalyzing a broad spectrum of reactions, particularly in chiral catalysis, with numerous applications reported in the synthesis of biologically relevant molecules and natural products [42,43,44].

**Scheme 1:**
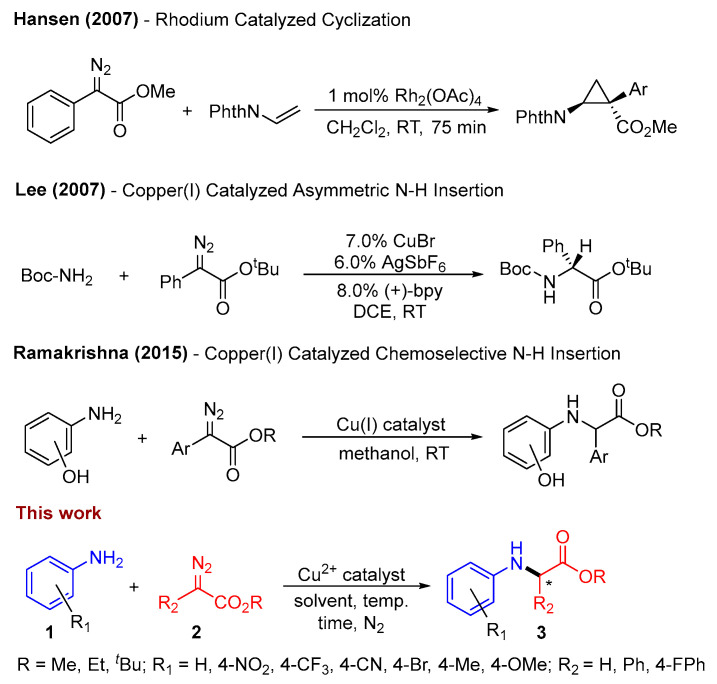
Previous work and our findings on the construction of C–N bonds [29,32,41]. * represent a chiral center formed in product.

Building upon these preceding findings (Scheme 1), we have developed a straightforward Schiff base ligand system for C–N bond construction, which employs a copper catalyst coordinated with a neutral tridentate ligand. This system offers a ligand architecture that can be tailored to enhance reactivity under sustainable conditions and create opportunities for selectivity. In this study, we present the synthesis of a (*E*)-1-(pyridin-2-yl)-*N*-(quinolin-8-yl)methanimine (PQM)-ligated copper(II) complex. We delve into the X-ray and electronic structure analysis of this complex and employ Cu^2+^(PQM) to catalytically facilitate C–N bond formation, yielding moderate to good yields. The investigation into reactivity sheds light on the mechanism underlying this process, providing a deeper understanding of the potential and limitations of this system as a catalyst for carbene transfer reactions.

## 2. Results and Discussion

**Scheme 2:**
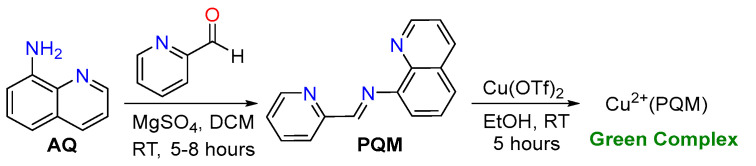
The synthesis of a Schiff base *NNN*-PQM ligand ligated with Cu(OTf)_2_.

The tridentate PQM ligand was synthesized in a one-step reaction based on its 8-aminoquinoline framework, as outlined in Scheme 2. The purified PQM ligand was coordinated with copper(II) to generate a green-colored Cu^2+^(PQM) complex, which was re-crystallized using a mixture of EtOH and DCM (1:6). 

**Figure 1:**
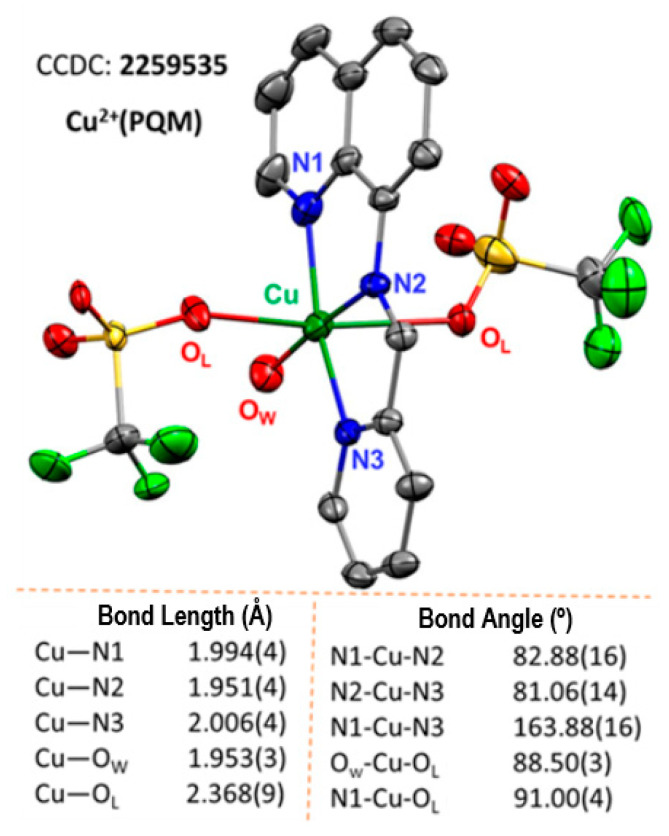
The copper complex of the Cu^2+^(PQM) crystal structure is shown as a 50% thermal ellipsoid plot. The H-atoms are removed for clarity. (Note: O_W_ = water, O_L_ = triflate anions, black = carbon, red = oxygen, blue = nitrogen, green = fluorine, yellow = sulfur).

The single-crystal XRD structure of Cu^2+^(PQM) was resolved (Figure 1). The Cu^2+^(PQM) complex was determined to possess two triflate ions and one water molecule in the unit cell. This 6-coordinate distorted octahedral complex shows the PQM ligand occupying the axial positions and a meridional binding mode. One bound water molecule completes the octahedral coordination sphere of this complex. The Cu–N (imine), Cu–N (quinoline), and Cu–N (pyridine) bond distances vary within the range of 1.951(4) Å–2.006(4) Å whereas the Cu–O (triflate) and Cu–O (water) bond distances are 2.398(9) Å and 1.953(3) Å, respectively. Curiously, the Cu–N bond from the imine is shorter than the other C–N bond from the quinoline and pyridine groups, showcasing the strong coordination mode of the complex. The tetragonal term *T* is defined as R_in_/R_out_ = 0.83 (where R_in_ = (1.994 + 1.951 + 2.006 + 1.953)/4 and R_out_ = 2.368), which indicates that the complex should be considered a tetragonally distorted octahedron [45,46,47]. The two triflate counter anions and one water molecule offer some opportunities for carbene or aniline coordination. Again, the substantially longer Cu–O bonding of the copper(II) triflate adducts implies that the Lewis acidity of the complex was well-modulated, which should influence the lability of these anions.

**Figure 2:**
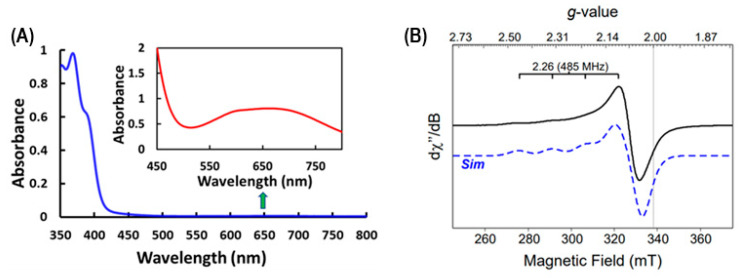
(**A**) UV–visible absorption spectrum of the Cu^2+^(PQM) at 5.0 µM in methanol (blue line) and the inset at 5.0 mM in methanol (red line), highlighting the low-intensity d→d transition. (**B**) X-band EPR spectrum of 4.0 mM Cu^2+^(PQM) shown as an experiment (black) and simulated (blue) data recorded at 10K.

The Cu^2+^(PQM) copper complex dissolved in methanol exhibits a broad, low-energy transition consistent with a d→d transition at 655 nm (ε_655_ = 161 M^−1^ cm^−1^), as shown in Figure 2A. The planar nature of the PQM ligand enforces two adjacent chelate rings, where both chelate rings are 5-membered. This observation is consistent with the five coordinated distorted square-pyramidal copper complexes having one PQM ligand and two bromide anions previously synthesized and reported [48]. The complex also exhibits two absorption bands in the UV region, which are likely attributable to the ligand π→π* or n→π* transitions associated with the aromatic rings in this copper complex. The electronic structure of the Cu^2+^(PQM) complex was characterized via electron paramagnetic resonance (EPR) spectroscopy, as illustrated in Figure 2B. Cu^2+^(PQM) indicates a distinct Cu^2+^ signal with a prototype hyperfine signal g_║_ = 2.07 and g_┴_ = 2.055, which is consistent with the nuclear spin associated with Cu (I = 3/2). This is an axial signal, and since g_║_ > g_┴_, the unpaired electron is likely distributed in the dx^2^-y^2^ orbital.

**Figure 3:**
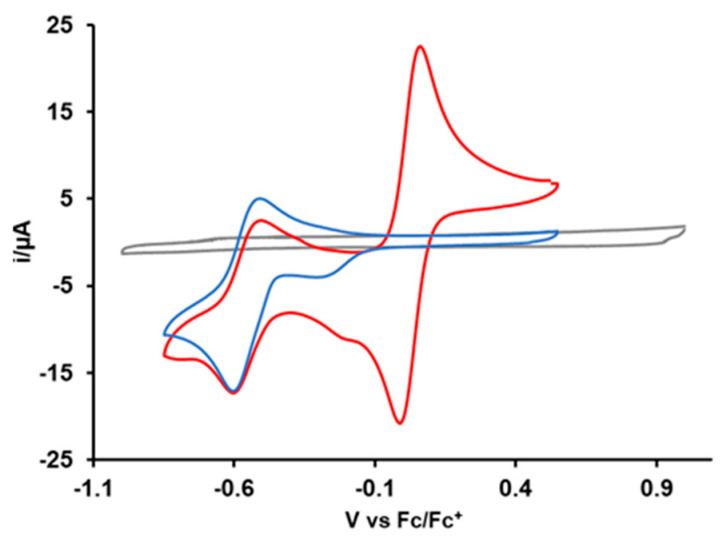
Cyclic voltammograms of solvent/acetonitrile (gray), 1.0 mM solution of Cu^2+^(PQM) complex (blue), and Cu^2+^(PQM) with ferrocene (red) at a scan rate of 0.1 V/s.

Figure 3 depicts the cyclic voltammogram of Cu^2+^(PQM). The CV exhibits a reversible redox wave at E_pa_ = −0.51 V and E_pc_ = −0.60 V and a chemically irreversible reduction peak at −0.26 V. The cathodic peak potential (E_pc_) value of −0.60 V indicates the reduction of Cu^2+^ to the Cu^+^ state. The half-wave potential (E_1/2_) value of −0.56 V vs. Fc^+^/Fc is the average potential of the anodic and cathodic peaks and is effectively equal to the standard reduction potential for the Cu^2+^/Cu^+^ complex. This E_1/2_ is consistent with Cu^2+^/Cu^+^ potentials associated with other copper(II) complexes chelated by *N*-donor ligands [13,49]. The irreversible reduction peak appearing in the CV data does not appear to be due to the decomposition of the complex, and the wave amplitude change with the scan rate indicates a diffusion-controlled mass transfer process (that is, not due to the adsorption of the complex to the electrode surface). This peak likely corresponds to a separate chemical species that exists at low concentrations in the solution.

The goal for generating this copper(II) complex was to explore its use as a catalyst for C–N bond-forming reactions. Our investigation began with the catalytic examination of the reaction between aniline (**1a**) and ethyl diazoacetate or EDA (**2a**), as shown in Table 1. We conducted the reaction using 10 mol% of simple copper salts, including CuCl, CuBr, Cu(OTf)_2_, CuCl_2_, CuBr_2_, and Cu(OAc)_2_. However, the conversions supported by these systems did not exceed 48% (Table 1, entries 1–6). However, the scope of these reactions demonstrates that the yields are highly influenced by the copper oxidation state, solvent medium, and temperature. The highest conversion was observed with copper(II) triflate, which is known to have activity towards diazo compounds [50]. When Cu^2+^(PQM) was employed as a catalyst, the conversion increased to 72% (Table 1, entry 7). Subsequently, the trials conducted in different solvents showed that DCM and DCE supported good conversions, while acetonitrile, methanol, and chlorobenzene exhibited low conversions (Table 1, entries 8–21). The extended reaction times also diminished the conversion (Table 1, entry 16). Interestingly, the use of 1.0 equivalent of acid or base resulted in reduced reaction yields (Table 1, entries 18 and 19). The reaction in buffer at pH 7.2 showed low conversion, which may be due to the low solubility of substrates in aqueous media. Finally, reducing the catalyst load to 5 and 2 mol% (Table 1 entry 22–23) led to a lower conversion.

**Scheme 3:**
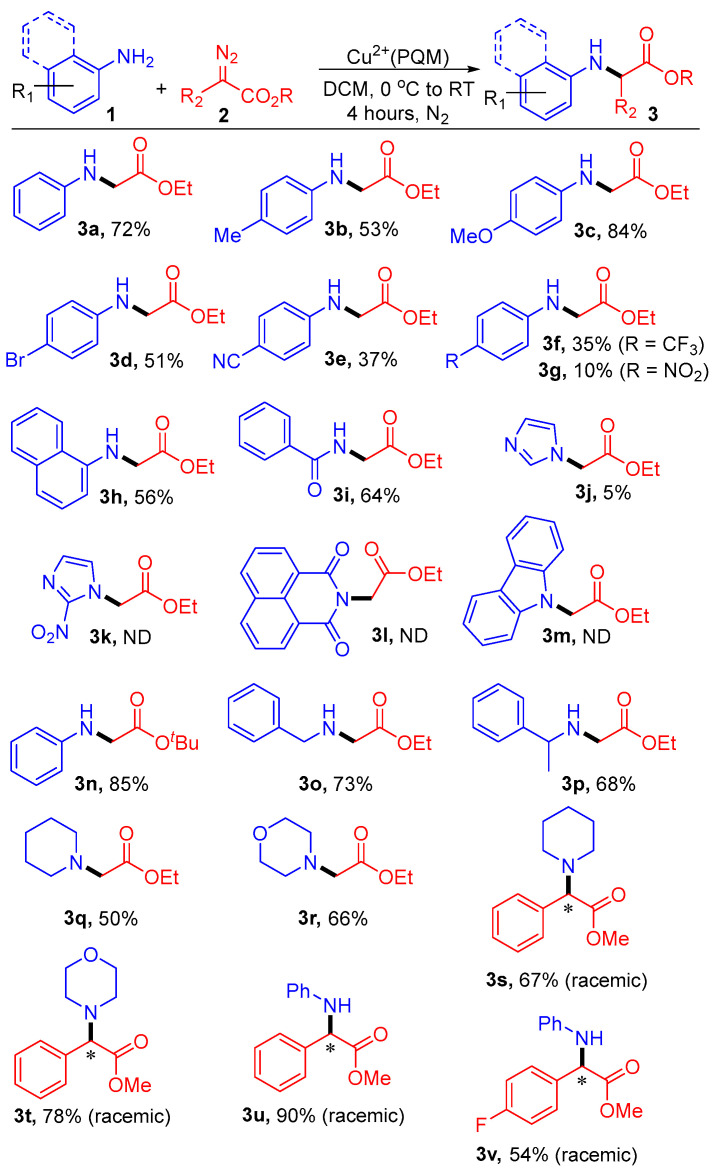
Substrate scopes of Cu^2+^(PQM)-catalyzed N–H insertion products. Reaction conditions: **1a** (0.5 mmol), **2a** (1.0 mmol), and Cu^2+^(PQM) (0.05 mmol) in 2.0 mL of DCM at 0 °C to RT for 4 h. The reaction conversions were determined through crude ^1^H-NMR spectroscopy using mesitylene as an internal standard. * represent a newly formed chiral centers in products, where reported conversions are a racemic mixture of both enantiomers.

Having established the optimal conditions, we proceeded to explore the substrate scope for the *N*-alkylation of various anilines and diazo compounds, as illustrated in Scheme 3. Substituted anilines and diazo compounds were successfully functionalized with good yields and a nearly 1:1 enantiomeric ratio. It was observed that with weak to strong electron-withdrawing groups (EWGs) in the para-position of the phenyl ring of aniline, there was reduced insertion, yielding the desired product in the range of 10% to 51% (**3d**–**3g**).

Conversely, the electron-donating groups (EDGs) provided good yields (**3b** and **3c**; 53% and 83%, respectively). 1-Naphthylamine and benzamide reacted with 2.0 equivalents of **2a**, yielding the corresponding products in yields of 56% and 64%, respectively (**3h** and **3i**). Unfortunately, imidazole, 2-nitroimidazole, 1,8-naphthalimide, and carbazole either did not react under our optimized conditions or yielded products in a yield of <5% (**3j**–**3m**). When the substrate Hammett parameters were plotted against the log(*k*_sub_/*k*_H_), there was a good linear correlation with a negative slope, where *k*_sub_ and *k*_H_ are the pseudo-first order rate constants for para-substituted aniline and aniline, respectively. The estimated rho (ρ) value for this correlation was negative and relatively small and should be interpreted with caution. Simply, this observation suggests a buildup of electron density on the coordinating aryl amine, which improves reaction efficiency where stronger Lewis basicity supports C–N bond-forming reactions. We also conducted experiments using the Cu^2+^(PQM) catalyst in the presence of substituted α-aryl-α-diazoesters and other substrates (**3s**–**v**). While the Cu^2+(^PQM) system showed strong reactivity in DCM, other copper complexes were described that favor transformations in MeOH or ionic media [34,35].

**Scheme 4:**
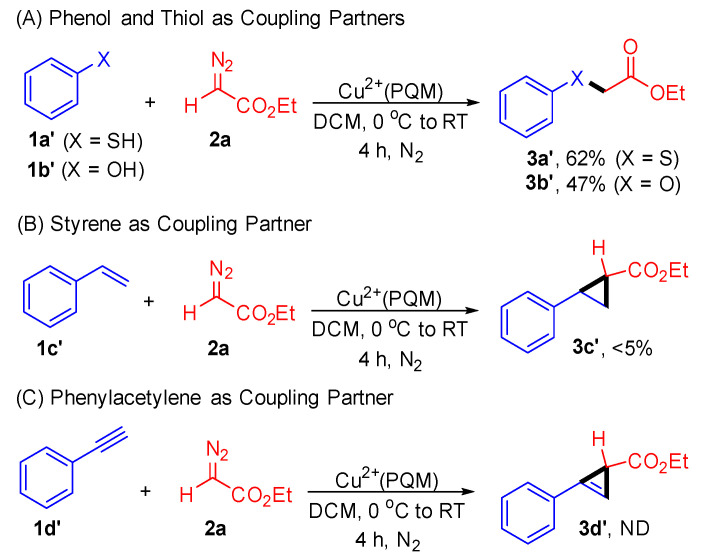
Control experiments for the carbene transfer reactions. Reaction conditions: **1** (0.5 mmol), **2a** (1.0 mmol), and Cu^2+^(PQM) (0.05 mmol) in 2.0 mL of DCM at 0 °C to RT for 4 h. The reaction conversations were determined through crude ^1^H-NMR spectroscopy using mesitylene as an internal standard.

Furthermore, we demonstrated the compatibility of this approach with phenol (**1a’**) and thiophenol (**1b’**) substrates. The reaction yielded C–O and C–S products with acceptable yields (Scheme 4). When investigating the reaction of styrene (**1c’**) and phenyl acetylene (**1d’**) with ethyl diazoacetate (**2a**) in the presence of the Cu^2+^(PQM) catalyst, product **3c’** was obtained in a yield of less than 5%, while product **3d’** was not detected. These results underscore the complementarity and orthogonality of the Cu^2+^(PQM) catalyst in facilitating carbene transfer reactions.

**Figure 4:**
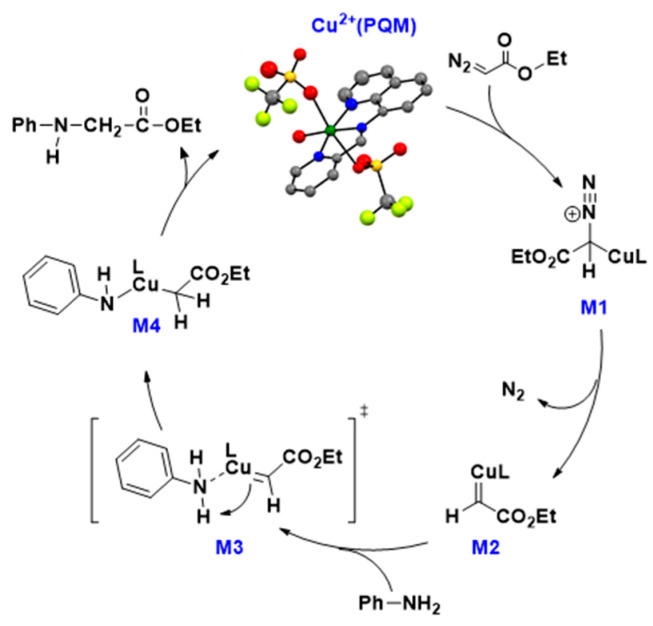
Proposed catalytic cycle for the carbene transfer reactions.

A generally accepted mechanism for C–N bond formation via diazo activation is shown in Figure 4. The proposed mechanism shows that the catalytic reaction occurs through five major steps. The first step of the reaction starts with water dissociation and diazo activation (shown here as **2a**) to generate a high-energy intermediate labeled here as M1. From this species, N_2_ is released, yielding the carbene compound M2.

The substate (shown here as aniline) reacts with the M2 compound via nucleophilic attack to create a relatively high-energy species (M3.) Based on unpublished data, we have drawn M3 as a transition state in this process. Significant energy stabilization occurs with proton transfer from aniline to protonate the carbene carbon, resulting in M4. Preliminary theoretical data supports M4 decaying through a high-energy transition state that generates the new C–N bond, releasing the reaction product and generating the resting Cu^2+^(PQM) species. The inductive and resonance effects observed in the catalytic trials suggest that the basicity and copper-modulated p*K*_a_ of the substrate likely impacts proton exchange in this mechanism (i.e., M2→M3→M4), which is consistent with this proton transfer event being the rate-determining step in this process.

## 3. Materials and Methods

8-Aminoquinoline, MgSO_4_, Cu(OTf)_2_, Cu(OAc)_2_ (Fisher Scientific, Waltham, MA, USA), methanol, acetone, dichloromethane (DCM), ethyl acetate, hexanes, chloroform-d (Sigma Aldrich, St. Louis, MO, USA), 2-pyridine carboxaldehyde, diazo compounds, and anilines (ACROS Organics, Bridgewater, MA, USA) were used as purchased without further purification. Silica gel from Alfa Aesar-Thermo Fisher Scientific (70–230 mesh) was used for flash chromatography. Unless otherwise stated, all the reagents were purchased from commercial sources.

### 3.1. Synthesis of 1-(Pyridin-2-yl)-N-(Quinolin-8-yl)Methanimine (PQM) Ligand and Resulting Copper(II) Complexes

The tridentate *NNN*-ligand (PQM) was generated via a one-step synthesis based on their 8-aminoquinoline frameworks. A 25-mL round-bottom (RB) flask was charged with a magnetic stir bar, 2.0 mmol of 8-aminoquinoline (AQ), 2.2 mmol of 2-pyridine carboxaldehyde, and 10.0 mmol of MgSO_4_. This mixture was solvated in 10.0 mL of dichloromethane (DCM) and the reaction was left to stir for approximately 5–8 h under an N_2_ atmosphere at room temperature and closed with a septum. After stirring, the reaction progress was monitored through TLC. Upon the completion of the precursors, the reaction mixture was filtered through celite. The evaporation of the solvent and subsequent purification via column chromatography on silica gel afforded the solvent removal of a light-yellow, oily product of PQM in a yield of 60%. This compound is known and the ^1^H-NMR data matches with reported spectra [51,52,53]. Yield 60% (320.0 mg), Yellow sticky liquid; ^1^H NMR (500 MHz, CDCl_3_) δ: 9.24 (d, *J* = 10.0 Hz, 1H), 9.01 (d, *J* = 4.0 Hz, 1H), 8.06 (dd, *J* = 2.0, 8.0 Hz, 1H), 7. 85 (d, *J* = 7.8 Hz, 1H), 7.66–7.40 (m, 4H), 7.19 (dd, *J* = 2.4, 7.6 Hz, 1H), 7.08 (dd, *J* = 2.4, 8.4 Hz, 1H), 6.90 (d, *J* = 8.4 Hz, 1H) ppm.

Cu^2+^(PQM) was prepared by stirring the synthesized PQM ligand with Cu(OTf)_2_ in ethanol at an ambient temperature for 5 h. The resulting Cu^2+^(PQM) complex exhibited a green color and was filtered and recrystallized using a mixture of EtOH and DCM (1:6). The FT-IR spectra of the Cu^2+^(PQM) complex showed distinctive stretching peaks of the azomethine group at approximately 1610 cm^−1^. This complex was characterized using powder and single-crystal X-ray diffraction studies.

### 3.2. General Procedure for C–N Bond Construction via Cu^2+^(PQM)-Catalyzed Redox-Neutral Type Cross-Coupling (**3a**–**3o**)

A 10-mL vial was charged with a magnetic stir bar, 0.5 mmol of anilines or related amines (**1a**–**1n**), 1.0 mmol of diazo-compounds (**2a**–**2c**), and 0.05 mmol of Cu^2+^(PQM). This mixture was solvated in 3.0 mL of dichloromethane (DCM) and the reaction was started at 0 °C to ambient temperature with stirring for approximately four hours under an N_2_ atmosphere. After the completion of the reaction time, the resulting reaction mixture was diluted with ethyl acetate and filtered through a thin pad of celite. The filtrate was evaporated under reduced pressure, and subsequent purification via column chromatography on silica gel afforded the desired products (**3a**–**3o**) obtained in the yields of roughly 90–10%. A summary of the characterization data for each known complex is provided in the Supplemental Information (SI) section associated with this work. The full characterization data is also included in the SI section for compound **3o**, including chiral HPLC traces, ^1^H, ^13^C, and ^19^F NMR data, and high-resolution mass spectrometry data.

### 3.3. Other Experimental Methods

All the catalytic C–N bond-forming reactions were conducted at 0 °C to room temperature using pre-dried glassware unless otherwise mentioned. The obtained products were characterized by melting points (m.p.), ^1^H NMR, ^13^C NMR, mass spectrometry (MS), and infrared spectra (IR). The ^1^H NMR and ^13^C NMR spectra were obtained on a Bruker 500 MHz, and the chemical shifts were reported in parts per million (ppm, δ) with CDCl_3_ as a reference. Based on the time-dependent trials, the reaction was defined as having first-order reaction kinetics. This implies that the rate of the reaction was proportional to the concentration of a single reactant, and the reaction’s progress could be mathematically described using first-order rate equations. HRMS-ESI mass data collection was conducted on an Orbitrap Exploris 240 mass spectrometer (Thermo Scientific, Waltham, MA, USA); ion source type: ESI in the positive mode; ionization method: infusion of individual compound (100 µM stock in MeOH) at a flow rate of 5 µL/min using syringe pump; source setting: spray voltage, 3500 V; ion transfer tube temperature, 275 °C; sheath gas pressure, 45 psi; aux gas pressure, 15 psi.

#### 3.3.1. Cyclic Voltammetry

The cyclic voltammetry (CV) analysis of the complex in a three-electrode cell was performed with Ar-degassed acetonitrile as the solvent and tert-butyl ammonium hexafluorophosphate as the supporting electrolyte. A 3 mm diameter glassy carbon electrode was the working equipment, a carbon rod was the counter, and a silver wire in silver nitrate/acetonitrile solution served as a quasi-reference electrode. The CVs were then referenced to the ferrocene/ferrocenium standard potential. The data were collected using a CHI 620A electrochemical analyzer. The CV peaks were analyzed using the eL-Chem viewer 3.3.

#### 3.3.2. Single-Crystal X-ray Crystallography

All the crystals selected for the X-ray crystallographic analysis were mounted on a cryo-loop using an oil cryoprotectant. The X-ray intensity data was measured at a low temperature [T = 120 K; Cu^2+^(PQM)] using three-circle goniometer geometry with a fixed Chie angle at = 54.74 deg Bruker AXS D8 Venture equipped with a Photon 100 CMOS active pixel sensor detector. Monochromatized Cu X-ray radiation (λ = 1.54178 Å) was selected for the measurement.

The X-ray intensity data was measured at a low temperature (T = 120K) using three-circle goniometer Kappa geometry with a fixed Kappa angle at = 54.74 deg Bruker AXS D8 Venture equipped with a Photon 100 CMOS active pixel sensor detector. Monochromatized Cu X-ray radiation (λ = 1.54178 Å) was selected for the measurement. All the frames were integrated with the aid of the Bruker SAINT software which is included in the package software: APEX4 v2021.10.0 using a narrow-frame algorithm. The integration of the data using a monoclinic unit cell yielded a total of 48,142 reflections to a maximum θ angle of 74.66° (0.80 Å resolution), of which 5136 were independent (average redundancy 9.373, completeness = 99.7%, R_int_ = 3.27%, R_sig_ = 1.79%) and 4785 (93.17%) were greater than 2σ (F^2^). The final cell constants of a = 24.0665(11) Å, b = 11.8732(5) Å, c = 17.8506(8) Å, β = 99.940(2) °, volume = 5024.2(4) Å^3^, were based upon the refinement of the XYZ-centroids of 1916 reflections above 20 σ (I) with 8.330° < 2θ < 149.7°. The data were corrected for absorption effects using the multi-scan method SADABS. The ratio of minimum to maximum apparent transmission was 0.741. The calculated minimum and maximum transmission coefficients (based on the crystal size) were 0.4300 and 0.5720. The structure was solved in a monoclinic unit cell; C-centered unit cell; space group: C 1 2/c 1 with Z = 8 for the formula unit, C17H19CuF6N3O10S2. Using the Bruker SHELXT software package, the refinement of the structure was carried out by least squares procedures on weighted F^2^ values using SHELXTL-2018/3 included in the APEX4 v2021 4.0 AXS Bruker program.

CCDC 2259535 contains the supplementary crystallographic data for this paper. These data can be obtained free of charge via http://www.ccdc.cam.ac.uk/conts/retrieving.html, accessed on 22 January 2024 (or from the CCDC, 12 Union Road, Cambridge CB2 1EZ, UK; Fax: +44-1223-336033; E-mail: deposit@ccdc.cam.ac.uk).

#### 3.3.3. UV–Vis Spectroscopy and Continuous Wave Electron Paramagnetic Resonance (CW EPR) Spectroscopy

UV–Vis spectroscopy was performed on a Shimadzu UV-2550 machine at room temperature. All UV–Vis data was collected in 1-cm quartz cuvettes in 5 × 10^−6^ M methanol solvent. Based on this data, the molar absorptivity of the complexes was calculated to be 161 M^−1^ cm^−1^.

CW EPR spectroscopy was performed on a Bruker ELEXSYS E540 X-band (Bruker-Biospin, Billerica, MA, USA). The liquid nitrogen spectra analysis was carried out using an unimodal resonator fixed with a Suprasil symmetric nitrogen dewar flask (Wilmad-Labglass, Vineland, NJ, USA), while the liquid helium spectra analysis was performed using an Oxford ESR900 cryostat and Oxford ITC 03 temperature controller.

CW EPR spectroscopy was performed on a Bruker ELEXSYS E540 X-band (Bruker-Biospin, Billerica, MA, USA). The liquid nitrogen spectra analysis was carried out using an unimodal resonator fixed with a Suprasil symmetric nitrogen dewar flask (Wilmad-Labglass), while the liquid helium spectra analysis was performed using an Oxford ESR900 cryostat and Oxford ITC 03 temperature controller. Simulations of the EPR spectra were completed using Spincount (6.5.7773.19293) developed by Professor Michael Hendrich at Carnegie Mellon University by utilizing the general spin Hamiltonian seen in Equation (1):

(1) $$\hat{H}=\beta_{e}{\overset{⃑}{B}}_{0}\cdot \tilde{g}\cdot \hat{S}+\hat{S}\cdot \tilde{A}\cdot \hat{I}$$

Here, $\tilde{g}$ is the g-tensor and $\tilde{A}$ is the nuclear hyperfine interaction, which is treated with the second-order perturbation theory [54,55]. The simulations take into consideration all intensity factors, both theoretical and experimental. The concentration of species can be used as a constraint during spectral simulation, which allows the quantitative determination of the concentration by comparing the experimental and simulated signal intensities [56]. The only unknown factor relating the spin concentration to the signal intensity is an instrumental factor that depends on the microwave detection system. This factor is determined using a copper(II) EDTA spin standard. The copper(II) complexes were dissolved in a solvent (acetonitrile or methanol) to obtain a final concentration of 3 to 4 mM. The solutions were then transferred into 3 mm quartz EPR tubes and frozen in liquid nitrogen. To remedy the broadening effects from aggregation, the Cu^2+^(PQM) sample was prepared in a binary mixture of 1:1 (*v*/*v*) toluene:acetonitrile for glassing. Unless noted elsewhere, the X-band (9.4 GHz) EPR spectra were collected at 10 and 77 K under non-saturating conditions.

## 4. Conclusions

In conclusion, a copper(II) complex, Cu^2+^(PQM), derived from an *NNN*-donor Schiff base ligand, was synthesized and characterized using various spectroscopic and structural techniques. This relatively simple planar tridentate ligand was assessed as part of the Cu^2+^(PQM) complex to catalyze the activation of diazo compounds and the resulting carbene transfer reaction to generate new C–N bonds. This reaction was optimized to catalyze C–N bond-forming processes with upwards of 84% conversions with select substrates. Remarkably, the C–N alkylated product was obtained in a single step without the need for additional base, oxidant, or additive, demonstrating the redox-neutral nature of the process. Some preference for carbene insertion in N–H bonds was achieved with a PQM-ligated copper catalyst over other O–H or S–H bonds. There are also clearly electronic and resonance effects that govern the productivity of this process over a range of substrates, where designing new catalysts aimed to overcome these tendencies remains a focus of our research group.

## Figures and Tables

**Table 1:** Copper-catalyzed C–N bond formation ^1^.

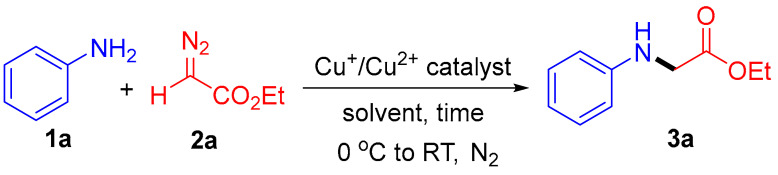			
Entry	Cu^+^/Cu^2+^ (mol %)	Solvent	Yields 3a % ^2^
1	CuCl (10)	DCM	20
2	CuBr (10)	DCM	15
3	Cu(OTf) (10)	DCM	48
4	CuCl_2_ (10)	DCM	ND
5	CuBr_2_ (10)	DCM	ND
6	Cu(OAc)_2_ (10)	DCM	38
7	Cu^2+^(PQM) (10)	DCM	72
8	Cu^2+^(PQM) (10)	DMSO	ND
9	Cu^2+^(PQM) (10)	MeOH	23
10	Cu^2+^(PQM) (10)	H_2_O	10
11	Cu^2+^(PQM) (10)	1,4-Dioxane	12
12	Cu^2+^(PQM) (10)	Chlorobenzene	20
13	Cu^2+^(PQM) (10)	CH_3_CN	20
14	Cu^2+^(PQM) (10)	DCM:MeOH	ND
15	Cu^2+^(PQM) (10)	DCM:H_2_O	23
16 ^3^	Cu^2+^(PQM) (10)	DCM	58
17	Cu^2+^(PQM) (10)	Buffer 7.2	12
18 ^4^	Cu^2+^(PQM) (10)	DCM	44
19 ^5^	Cu^2+^(PQM) (10)	DCM	38
20	Cu^2+^(PQM) (10)	*o*DCB	ND
21	Cu^2+^(PQM) (10)	DCE	57
22	Cu^2+^(PQM) (5)	DCM	68
23	Cu^2+^(PQM) (2)	DCM	60

^1^ Reaction conditions: **1a** (0.5 mmol), **2a** (1.0 mmol), and Cu^2+^(PQM) (0.05 mmol) in 2.0 mL of DCM at 0 °C to room temperature (RT) for 4 h. ^2^ The reaction yields were determined through crude ^1^H-NMR spectroscopy using mesitylene as an internal standard. ^3^ Reaction performed for 24 h. ^4^ NaOAc was used as a base. ^5^ AcOH was used as an additive.

## Data Availability

The data presented in this study are available on request from the corresponding author.

## Supplementary Materials

The following supporting information can be downloaded at: https://www.mdpi.com/article/10.3390/molecules29030730/s1, General synthetic procedures; Figure S1: Cu^2+^(PQM) LeBail refinement fitting profile of PXRD and single-XRD; Figure S2: Cu^2+^(PQM) complex crystalline phase PXRD; General catalytic procedures; Tabulated Spectroscopic data; Figure S3: HPLC spectra of compound **3o** and integration data; Figure S4: ^1^H NMR spectra of compound **3o**; Figure S5: ^13^C NMR spectra of compound **3o**; Figure S6: ^19^F NMR spectra of compound **3o**; Figure S7: HRMS data for the compound **3o**; General kinetic procedures; Figure S8: N–H insertion reaction kinetics plot for compound **3a**; Figure S9: N–H insertion reaction kinetics plot for compound **3b**; Figure S10: N–H insertion reaction kinetics plot for compound **3c**; Figure S11: N–H insertion reaction kinetics plot for compound **3d**; Figure S12: N–H insertion reaction kinetics plot for compound **3e**; Figure S13: N–H insertion reaction kinetics plot for compound **3f**; Figure S14: N–H insertion reaction kinetics plot for compound **3g**; Figure S15: Cu^2+^(PQM) complex crystal view on mounted on a cryoLoop. Figure S16: Crystallographic data, structure of Cu^2+^(PQM) complex, and related report; Table S1: Sample and crystal data for Cu^2+^(PQM) complex; Table S2: Data collection and structure refinement for Cu^2+^(PQM) complex. References [57,58,59,60,61,62,63,64,65,66,67,68,69,70] are cite in the Supplementary Materials.

## References

[1] Itoh S. (2015). Developing Mononuclear Copper–Active-Oxygen Complexes Relevant to Reactive Intermediates of Biological Oxidation Reactions. Acc. Chem. Res..

[2] Suzuki M. (2007). Ligand Effects on Dioxygen Activation by Copper and Nickel Complexes: Reactivity and Intermediates. Acc. Chem. Res..

[3] Pelosi G., Bisceglie F., Bignami F., Ronzi P., Schiavone P., Re M.C., Casoli C., Pilotti E. (2010). Antiretroviral Activity of Thiosemicarbazone Metal Complexes. J. Med. Chem..

[4] Gou Y., Chen M., Li S., Deng J., Li J., Fang G., Yang F., Huang G. (2021). Dithiocarbazate-Copper Complexes for Bioimaging and Treatment of Pancreatic Cancer. J. Med. Chem..

[5] Bormio Nunes J.H., Hager S., Mathuber M., Pósa V., Roller A., Enyedy É.A., Stefanelli A., Berger W., Keppler B.K., Heffeter P. (2020). Cancer Cell Resistance Against the Clinically Investigated Thiosemicarbazone COTI-2 Is Based on Formation of Intracellular Copper Complex Glutathione Adducts and ABCC1-Mediated Efflux. J. Med. Chem..

[6] Tardito S., Barilli A., Bassanetti I., Tegoni M., Bussolati O., Franchi-Gazzola R., Mucchino C., Marchiò L. (2012). Copper-Dependent Cytotoxicity of 8-Hydroxyquinoline Derivatives Correlates with Their Hydrophobicity and Does Not Require Caspase Activation. J. Med. Chem..

[7] Santini C., Pellei M., Gandin V., Porchia M., Tisato F., Marzano C. (2014). Advances in Copper Complexes as Anticancer Agents. Chem. Rev..

[8] Karlin K.D., Gultneh Y. (1985). Bioinorganic Chemical Modeling of Dioxygen-Activating Copper Proteins. J. Chem. Educ..

[9] Lewis E.A., Tolman W.B. (2004). Reactivity of Dioxygen-Copper Systems. Chem. Rev..

[10] Hu Q.-Q., Su X.-J., Zhang M.-T. (2018). Electrocatalytic Water Oxidation by an Unsymmetrical Di-Copper Complex. Inorg. Chem..

[11] Shimizu D., Kurose A., Nishikata T. (2022). Remote Nucleophilic Substitution at a C(sp^3^)–H Bond of α-Bromocarboxamides via 1,4-Hydrogen Atom Transfer To Access N-Acyl-N,O-Acetal Compounds. Org. Lett..

[12] Zhang Z., Górski B., Leonori D. (2022). Merging Halogen-Atom Transfer (XAT) and Copper Catalysis for the Modular Suzuki–Miyaura-Type Cross-Coupling of Alkyl Iodides and Organoborons. J. Am. Chem. Soc..

[13] Cope J.D., Valle H.U., Hall R.S., Riley K.M., Goel E., Biswas S., Hendrich M.P., Wipf D.O., Stokes S.L., Emerson J.P. (2020). Tuning the Copper(II)/Copper(I) Redox Potential for More Robust Copper-Catalyzed C–N Bond Forming Reactions. Eur. J. Inorg. Chem..

[14] Cope J.D., Sheridan P.E., Galloway C.J., Awoyemi R.F., Stokes S.L., Emerson J.P. (2020). Synthesis and Characterization of a Tetradentate, N-Heterocyclic Carbene Copper(II) Complex and Its Use as a Chan–Evans–Lam Coupling Catalyst. Organometallics.

[15] Raju S., Sheridan P.E., Hauer A.K., Garrett A.E., McConnell D.E., Thornton J.A., Stokes S.L., Emerson J.P. (2022). Cu-Catalyzed Chan–Evans–Lam Coupling Reactions of 2-Nitroimidazole with Aryl Boronic Acids: An Effort toward New Bioactive Agents against S. Pneumoniae. Chem. Biodivers..

[16] Adhikari B., Teimouri M., Akin J.W., Raju S., Stokes S.L., Emerson J.P. (2023). Cu−NHC Complex for Chan-Evans-Lam Cross-Coupling Reactions of N-Heterocyclic Compounds and Arylboronic Acids. Eur. J. Org. Chem..

[17] Hassan J., Sévignon M., Gozzi C., Schulz E., Lemaire M. (2002). Aryl−Aryl Bond Formation One Century after the Discovery of the Ullmann Reaction. Chem. Rev..

[18] Evano G., Blanchard N., Toumi M. (2008). Copper-Mediated Coupling Reactions and Their Applications in Natural Products and Designed Biomolecules Synthesis. Chem. Rev..

[19] Allen S.E., Walvoord R.R., Padilla-Salinas R., Kozlowski M.C. (2013). Aerobic Copper-Catalyzed Organic Reactions. Chem. Rev..

[20] Guo X.-X., Gu D.-W., Wu Z., Zhang W. (2015). Copper-Catalyzed C–H Functionalization Reactions: Efficient Synthesis of Heterocycles. Chem. Rev..

[21] Maestre L., Ozkal E., Ayats C., Beltrán Á., Díaz-Requejo M.M., Pérez P.J., Pericàs M.A. (2015). A Fully Recyclable Heterogenized Cu Catalyst for the General Carbene Transfer Reaction in Batch and Flow. Chem. Sci..

[22] Kroitor A.P., Cailler L.P., Martynov A.G., Gorbunova Y.G., Tsivadze A.Y., Sorokin A.B. (2017). Unexpected Formation of a μ-Carbido Diruthenium(Iv) Complex during the Metalation of Phthalocyanine with Ru3(CO)12 and Its Catalytic Activity in Carbene Transfer Reactions. Dalt. Trans..

[23] Chen J.-Q., Li J.-H., Dong Z.-B. (2020). A Review on the Latest Progress of Chan-Lam Coupling Reaction. Adv. Synth. Catal..

[24] Seifinoferest B., Tanbakouchian A., Larijani B., Mahdavi M. (2021). Ullmann-Goldberg and Buchwald-Hartwig C−N Cross Couplings: Synthetic Methods to Pharmaceutically Potential N-Heterocycles. Asian J. Org. Chem..

[25] Hari D.P., Waser J. (2016). Copper-Catalyzed Oxy-Alkynylation of Diazo Compounds with Hypervalent Iodine Reagents. J. Am. Chem. Soc..

[26] Durka J., Turkowska J., Gryko D. (2021). Lightening Diazo Compounds?. ACS Sustain. Chem. Eng..

[27] Dong S., Liu X., Feng X. (2022). Asymmetric Catalytic Rearrangements with α-Diazocarbonyl Compounds. Acc. Chem. Res..

[28] Ford A., Miel H., Ring A., Slattery C.N., Maguire A.R., McKervey M.A. (2015). Modern Organic Synthesis with α-Diazocarbonyl Compounds. Chem. Rev..

[29] Lee E.C., Fu G.C. (2007). Copper-Catalyzed Asymmetric N−H Insertion Reactions:  Couplings of Diazo Compounds with Carbamates to Generate α-Amino Acids. J. Am. Chem. Soc..

[30] Xiang Y., Wang C., Ding Q., Peng Y. (2019). Diazo Compounds: Versatile Synthons for the Synthesis of Nitrogen Heterocycles via Transition Metal-Catalyzed Cascade C–H Activation/Carbene Insertion/Annulation Reactions. Adv. Synth. Catal..

[31] Khanal H.D., Thombal R.S., Maezono S.M.B., Lee Y.R. (2018). Designs and Strategies for the Halo-Functionalization of Diazo Compounds. Adv. Synth. Catal..

[32] Melby T., Hughes R.A., Hansen T. (2007). H. Diastereoselective Synthesis of 1-Aryl-2-Amino-Cyclopropane Carboxylates. Synlett.

[33] Cailler L.P., Kroitor A.P., Martynov A.G., Gorbunova Y.G., Sorokin A.B. (2021). Selective Carbene Transfer to Amines and Olefins Catalyzed by Ruthenium Phthalocyanine Complexes with Donor Substituents. Dalt. Trans..

[34] Kantam M.L., Laha S., Yadav J., Jha S. (2009). Nanocrystalline Copper(II) Oxide Catalyzed Aza-Michael Reaction and Insertion of α-Diazo Compounds into N–H Bonds of Amines. Tetrahedron Lett..

[35] Kantam M.L., Neelima B., Reddy C.V. (2006). Cu(Acac)2 Immobilized in Ionic Liquids: A Reusable Catalytic System for the Insertion of α-Diazo Compounds into NH Bonds of Amines. J. Mol. Catal. A Chem..

[36] Han F., Choi P.H., Ye C.-X., Grell Y., Xie X., Ivlev S.I., Chen S., Meggers E. (2022). Cyclometalated Chiral-at-Ruthenium Catalyst for Enantioselective Ring-Closing C(sp^3^)–H Carbene Insertion to Access Chiral Flavanones. ACS Catal..

[37] Tanbouza N., Keipour H., Ollevier T. (2019). FeII-Catalysed Insertion Reaction of α-Diazocarbonyls into X–H Bonds (X = Si, S, N, and O) in Dimethyl Carbonate as a Suitable Solvent Alternative. RSC Adv..

[38] Li Y., Zhao Y.-T., Zhou T., Chen M.-Q., Li Y.-P., Huang M.-Y., Xu Z.-C., Zhu S.-F., Zhou Q.-L. (2020). Highly Enantioselective O–H Bond Insertion Reaction of α-Alkyl- and α-Alkenyl-α-Diazoacetates with Water. J. Am. Chem. Soc..

[39] Liu B., Zhu S.-F., Zhang W., Chen C., Zhou Q.-L. (2007). Highly Enantioselective Insertion of Carbenoids into N−H Bonds Catalyzed by Copper Complexes of Chiral Spiro Bisoxazolines. J. Am. Chem. Soc..

[40] Nam D., Tinoco A., Shen Z., Adukure R.D., Sreenilayam G., Khare S.D., Fasan R. (2022). Enantioselective Synthesis of α-Trifluoromethyl Amines via Biocatalytic N–H Bond Insertion with Acceptor-Acceptor Carbene Donors. J. Am. Chem. Soc..

[41] Ramakrishna K., Murali M., Sivasankar C. (2015). Chemoselective Carbene Insertion into the N–H Bond over O–H Bond Using a Well-Defined Single Site (P–P)Cu(I) Catalyst. Org. Lett..

[42] Zhao X., Zhang Y., Wang J. (2012). Recent Developments in Copper-Catalyzed Reactions of Diazo Compounds. Chem. Commun..

[43] Álvarez M., Besora M., Molina F., Maseras F., Belderrain T.R., Pérez P.J. (2021). Two Copper-Carbenes from One Diazo Compound. J. Am. Chem. Soc..

[44] Namitharan K., Zhu T., Cheng J., Zheng P., Li X., Yang S., Song B.-A., Chi Y.R. (2014). Metal and Carbene Organocatalytic Relay Activation of Alkynes for Stereoselective Reactions. Nat. Commun..

[45] Efthimiadou E.K., Katsaros N., Karaliota A., Psomas G. (2007). Synthesis, Characterization, Antibacterial Activity, and Interaction with DNA of the Vanadyl-Enrofloxacin Complex. Bioorg. Med. Chem. Lett..

[46] Ramakrishnan S., Palaniandavar M. (2008). Interaction of *rac*-[Cu(Diimine)_3_]^2+^ and *rac*-[Zn(Diimine)_3_]^2+^ Complexes with CT DNA: Effect of Fluxional Cu(II) Geometry on DNA Binding, Ligand–Promoted Exciton Coupling and Prominent DNA Cleavage. Dalt. Trans..

[47] Murphy B., Aljabri M., Ahmed A.M., Murphy G., Hathaway B.J., Light M.E., Geilbrich T., Hursthouse M.B. (2006). Structural Systematics of the [Cu(Chelate)_3_][Y]_2_ Series. An Interesting Crystallographic Structural Insight Involving Vibronic Coupling and the Jahn–Teller Effect (JTE). The Syntheses and Low Temperature Crystal Structures of Tris(2,2′bipyridyl)Copper(II) Tetraphenylborate and Tris(2,2′bipyridyl)Zinc(II) Tetraphenylborate. Dalt. Trans..

[48] Lu J., Sun Q., Li J.-L., Jiang L., Gu W., Liu X., Tian J.-L., Yan S.-P. (2014). Two Water-Soluble Copper(II) Complexes: Synthesis, Characterization, DNA Cleavage, Protein Binding Activities and in Vitro Anticancer Activity Studies. J. Inorg. Biochem..

[49] Valle H.U., Riley K.M., Russell D.E., Wolgemuth D.K., Voges-Haupt C.F., Rogers T.A., Redd S.L., Stokes S.L., Emerson J.P. (2018). Synthesis, Characterization, and Structure of a [Cu(Phen)_2_(OTf)]OTf Complex: An Efficient Nitrogen Transfer Pre-Catalyst. ChemistrySelect.

[50] Doyle M.P. (1986). Catalytic Methods for Metal Carbene Transformations. Chem. Rev..

[51] Lions F., Martin K.V. (1957). Tridentate Chelate Compounds. I. J. Am. Chem. Soc..

[52] Kinunda G., Jaganyi D. (2014). Understanding the Electronic and π-Conjugation Roles of Quinoline on Ligand Substitution Reactions of Platinum(II) Complexes. Transit. Met. Chem..

[53] Sorouraddin M.-H., Rashidi M.-R., Shabani B., Ghorbani-Kalhor E. (2005). Spectrofluorimetric Determination of Cu^2+^ Using New Fluorogenic Reagent. Chin. J. Chem..

[54] Palmer G., Que L.J. (2000). Electron Paramagnetic Resonance of Metalloproteins. Physical Methods in Bioinorganic Chemistry Spectroscopy and Magnetism.

[55] Hales B.J., Scott R.A., Lukehart C.M. (2007). Electron Paramagnetic Resonance (EPR) Spectroscopy. Applications of Physical Methods to Inorganic and Bioinorganic Chemistry.

[56] Petasis D.T., Hendrich M.P., Qin P.Z., Warncke K. (2015). Chapter Eight—Quantitative Interpretation of Multifrequency Multimode EPR Spectra of Metal Containing Proteins, Enzymes, and Biomimetic Complexes. Methods in Enzymology.

[57] Quach T.D., Batey R.A. (2003). Ligand- and Base-Free Copper(II)-Catalyzed C−N Bond Formation:Cross-Coupling Reactions of Organoboron Compounds with Aliphatic Amines and Anilines. Org. Lett..

[58] Sreenilayam G., Fasan R. (2015). Myoglobin-Catalyzed Intermolecular Carbene N-H Insertion with Arylamine Substrates. Chem Comm..

[59] Ouyang L., Li J., Zheng J., Huang J., Qi C., Wu W., Jiang H. (2017). Access to α-Amino Acid Esters through Palladium-Catalyzed Oxidative Amination of Vinyl Ethers with Hydrogen Peroxide as the Oxidant and Oxygen Source. Angew. Chem. Int. Ed..

[60] Aviv I., Gross Z. (2008). Iron(III) Corroles and Porphyrins as Superior Catalysts for the Reactions of Diazoacetates with Nitrogen- or Sulfur-Containing Nucleophilic Substrates: Synthetic Uses and Mechanistic Insights. Chem. A Eur. J..

[61] Rohlmann R., Stopka T., Richter H., García Mancheño O. (2013). Iron-Catalyzed Oxidative Tandem Reactions with TEMPO Oxoammonium Salts: Synthesis of Dihydroquinazolines and Quinolines. J. Org. Chem..

[62] Wolf M.W., Vargas D.A., Lehnert N. (2017). Engineering of RuMb: Toward a Green Catalyst for Carbene Insertion Reactions. Inorg. Chem..

[63] Abu-Elfotoh A.-M. (2017). NH Insertion Reactions Catalyzed by Reusable Water-Soluble Ruthenium(II)-Hm-Phenyloxazoline Complex. Tetrahedron Lett..

[64] Ueda T., Konishi H., Manabe K. (2012). Trichlorophenyl Formate: Highly Reactive and Easily Accessible Crystalline CO Surrogate for Palladium-Catalyzed Carbonylation of Aryl/Alkenyl Halides and Triflates. Org. Lett..

[65] Hattori G., Sakata K., Matsuzawa H., Tanabe Y., Miyake Y., Nishibayashi Y. (2010). Copper-Catalyzed Enantioselective Propargylic Amination of Propargylic Esters with Amines: Copper−Allenylidene Complexes as Key Intermediates. J. Am. Chem. Soc..

[66] Ugarriza I., Uria U., Carrillo L., Vicario J.L., Reyes E. (2014). Base-Promoted C→N Acyl Rearrangement:An Unconventional Approach to α-Amino Acid Derivatives. Chem. A Eur. J..

[67] Steck V., Sreenilayam G., Gillingham D., Fei N., Moody C., Zhu S., Zhou Q., Yates P., Moyer M., Feldman P. (2020). Selective Functionalization of Aliphatic Amines via Myoglobin-Catalyzed Carbene N–H Insertion. Synlett.

[68] Baumann L.K., Mbuvi H.M., Du G., Woo L.K. (2007). Iron Porphyrin Catalyzed N−H Insertion Reactions with Ethyl Diazoacetate. Organometallics.

[69] Miura T., Morimoto M., Murakami M. (2012). Copper-Catalyzed Amination of Silyl Ketene Acetals with N-Chloroamines. Org. Lett..

[70] Deng Q.-H., Xu H.-W., Yuen A.W.-H., Xu Z.-J., Che C.-M. (2008). Ruthenium-Catalyzed One-PotCarbenoid N−H Insertion Reactions and Diastereoselective Synthesis of Prolines. Org. Lett..

