# Supplementary material for: Aminoquinoline-Based Tridentate (NNN)-Copper Catalyst for C–N Bond-Forming Reactions from Aniline and Diazo Compounds
Source: Molecules. 2024 Feb 5;29(3):730. doi: 10.3390/molecules29030730 (PMC10856582; doi:10.3390/molecules29030730)
Supplement: Supplementary file 1 [file molecules-29-00730-s001.zip › molecules-2808003-supplementary.pdf]

Supplementary Information

Aminoquinoline-Based Tridentate (*NNN*)-Copper Catalyst for C–N Bond-Forming Reactions from Aniline and Diazo Compounds

Mohsen Teimouri [1], Selvam Raju [1], Edward Acheampong [1], Allison N. Schmittou [2], Bruno Donnadieu [1], David O. Wipf [1], Brad S. Pierce [2], Sean L. Stokes [1] and Joseph P. Emerson [1,*]

[1] Department of Chemistry, Mississippi State University, Starkville, MS 39762, USA; bdonnadieu@chemistry.msstate.edu (B.D.); dwipf@chemistry.msstate.edu (D.O.W.)
[2] Department of Chemistry and Biochemistry, The University of Alabama, 3097D Shelby Hall, Tuscaloosa, AL 35487, USA
* Correspondence: jemerson@chemistry.msstate.edu; Tel.: +1-662-325-4633

Table of Contents

Procedure for the synthesis of PQM ligand	1
Procedure for the synthesis of PQM ligated copper complex	2
Powder X-ray diffraction (PXRD) analysis for the Cu^{2+}(PQM) fitted with the LeBail profile fitting.	3-4
General procedure for C–N bond construction by Cu^{2+}(PQM) catalyzed redox-neutral type cross-coupling (**3a**-**3v**)	5-10
HPLC spectra of compound **3v**	11
(^{1}H, ^{13}C and ^{19}F) NMR spectra of compound **3v**	12-14
HRMS data for the compound **3v**	15
Cu^{2+}(PQM) catalyst N–H insertion reaction kinetics plot	16-22
Cu^{2+}(PQM) complex crystal data	23-25
References	26

Procedure for the synthesis of PQM ligand

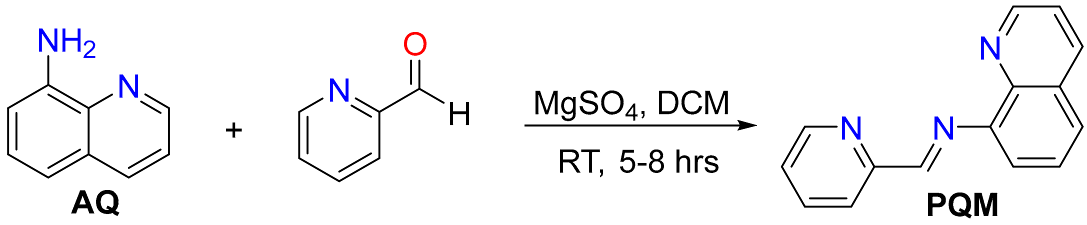

A 25-mL round-bottom (RB) flask was charged with a magnetic stir bar, 2.0 mmol of 8-aminoquinoline (**AQ**), 2.2 mmol of 2-pyridine carboxaldehyde, and 10.0 mmol of $MgSO_4$. This mixture was solvated in 10.0 mL of dichloromethane (DCM) and the reaction was left to stir for approximately 5-8 hours under an N_2 atmosphere at room temperature in a closed with a septum. After stirring, the reaction progress was monitored through TLC. Upon the completion of the precursors, the reaction mixture was filtered through celite. Evaporation of the solvent and subsequent purification by column chromatography on silica gel afforded the oily product of **PQM** in 60% yields. This compound is known and ^{1}H-NMR data matches with reported spectra.[1–3] Yield 60% (320.0 mg), Yellow sticky liquid; ^{1}H NMR (500 MHz, $CDCl_3$) δ: 9.24 (d, J = 10.0 Hz, 1H), 9.01 (d, J = 4.0 Hz, 1H), 8.06 (dd, J = 2.0, 8.0 Hz, 1H), 7. 85 (d, J = 7.8 Hz, 1H), 7.66–7.40 (m, 4H), 7.19 (dd, J = 2.4, 7.6 Hz, 1H), 7.08 (dd, J = 2.4, 8.4 Hz, 1H), 6.90 (d, J = 8.4 Hz, 1H) ppm.

Procedure for the synthesis of PQM ligated copper complex

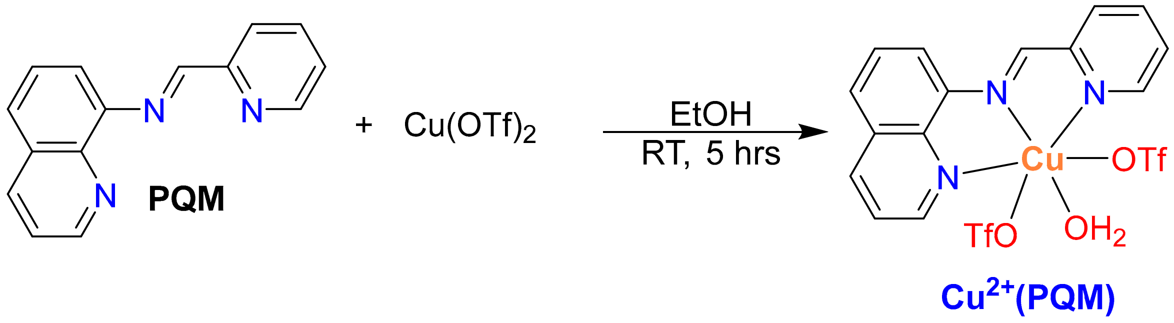

A 25-mL round-bottom (RB) flask was equipped with a stirring bar and charged with 1.0 mmol of **PQM**, 1.0 mmol of $Cu(OTf)_2$, and solvated with 5.0 mL of EtOH solvent. The mixture was stirred at ambient temperature for 6h, and then EtOH was removed *in vacuo*. After, the precipitate was washed with methanol to afford the Cu^{2+}(PQM) in green color powder. The Cu^{2+}(PQM) could be crystallized in EtOH and DCM. Crystallization and evaporation of the solvent afforded the green color product of Cu^{2+}(PQM) in 71% yield. Anal. calcd for $C_{17}H_{13}CuF_6N_3O_7S_2$: C, 33.31; H, 2.14; N, 6.86; S, 10.46. Found: C, 31.46; H, 2.16; N, 6.54; S, 12.46.

Powder X-ray diffraction (PXRD) analysis for the Cu^{2+}(PQM) fitted with the LeBail profile fitting.

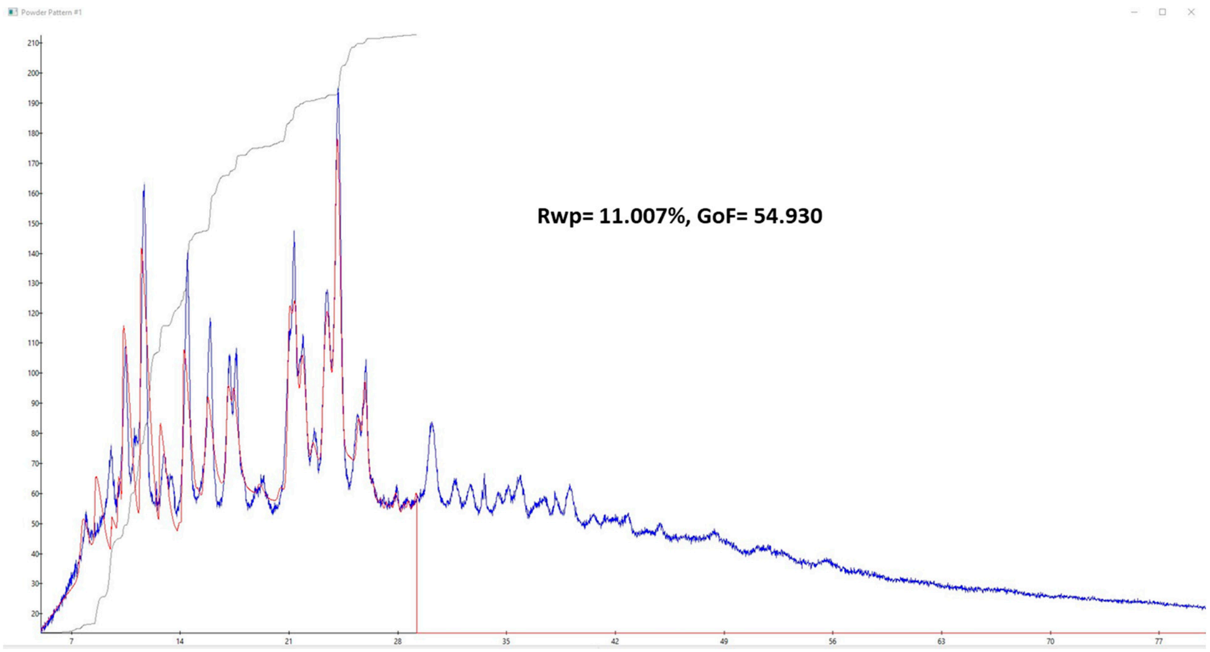

Figure S1. Cu^{2+}(PQM) LeBail refinement fitting profile of PXRD and single-XRD [Blue– measured and Red – stimulated].

**Powder sample:**	**Single crystal:**
Symmetry_space_group_name_H-M 'C2/m'	Symmetry_space_group_name_H-M ' C2/m '
a = 21.95385 Å	a = 24.0665 Å
b = 10.95825 Å	b = 11.8732 Å
c =17.17444Å	c = 17.8506 Å
β = 101.089°	β = 99.940°
volume = 4054.60 $Å^3$	volume = 5024.2 $Å^3$

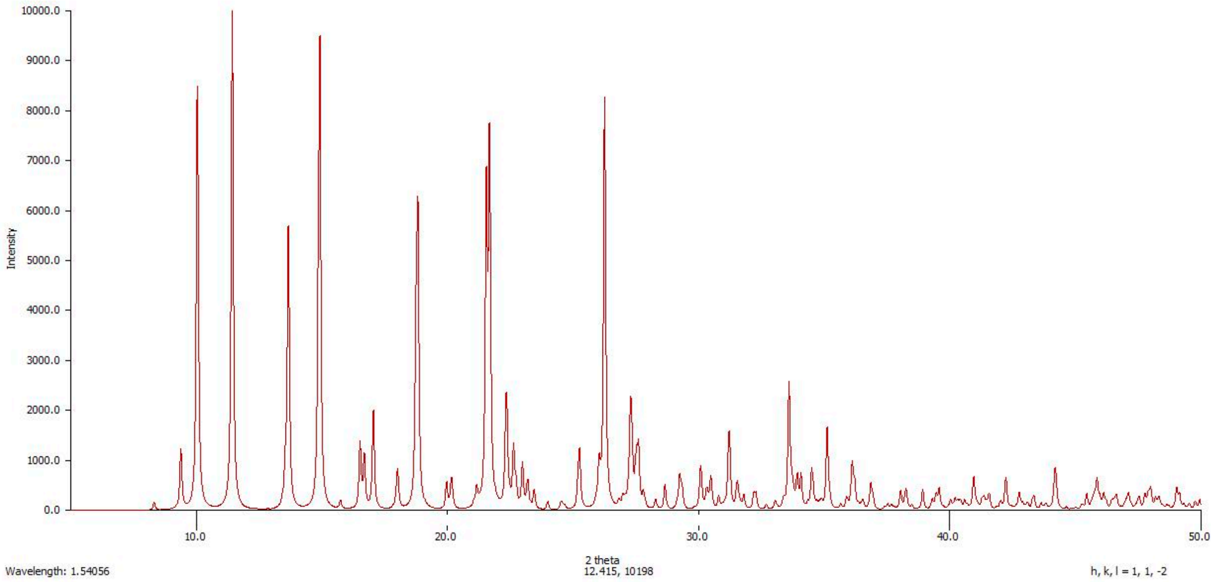

CRYSTALLINITY REPORT

% crystallinity: 69.13
% amorphous: 30.87
II crystallinity: 449.7
II amorphous: 200.8

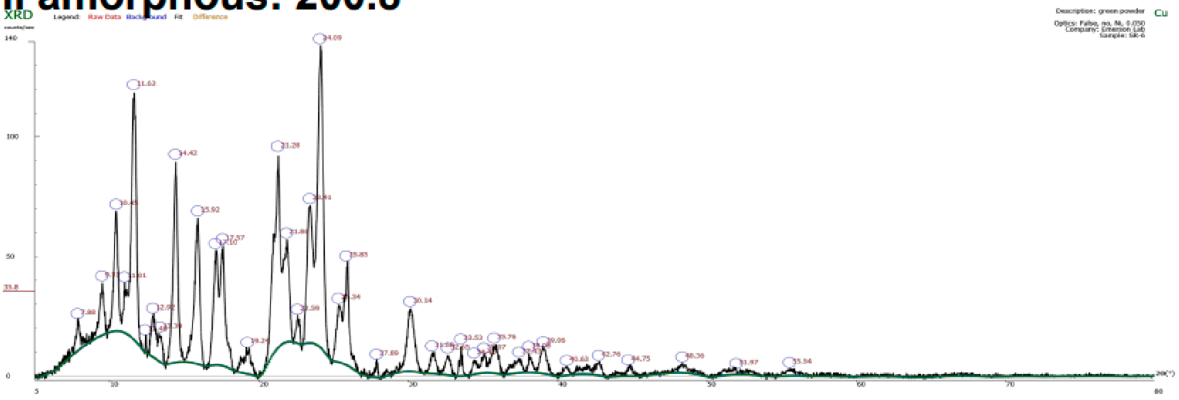

2th Range:	5.00° ... 80.00°
delta 2th:	0.0197°
Dwell:	4 sec
Scan time:	00d 03h 11m 23s
HV:	30kV, 20mA

Figure S2. Cu^{2+}(PQM) complex crystalline phase PXRD.

General procedure for C–N bond construction by Cu^{2+}(PQM) catalyzed redox-neutral type cross-coupling (3a-3v)

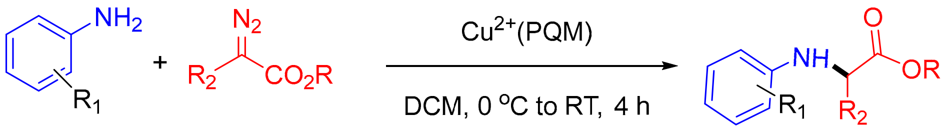

A 10-mL vial was charged with a magnetic stir bar, 0.5 mmol of amine, 1.0 mmol of diazo-compound, and 0.05 mmol of Cu^{2+}(PQM). This mixture was solvated in 3.0 mL of dichloromethane (DCM) and the reaction was started at 0 °C to ambient temperature stir for approximately four hours under an N_2 atmosphere. After the completion of the reaction time, the resulting reaction mixture was diluted with ethyl acetate and filtered through a thin pad of celite. The filtrate was evaporated under reduced pressure and subsequent purification by column chromatography on silica gel afforded the desired products.

Spectroscopic and physical data of all compounds (3a-3v)

Ethyl phenylglycinate (3a)[4]

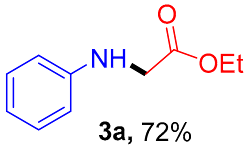

3a, 72%

Yield: 72%; Pale browish sticky solid; mp = 58 °C; ^{1}H NMR (500 MHz, $CDCl_3$) δ: 7.21 (t, J = 4.5 HZ, 2H), 6.74 (t, J = 7.2 Hz, 1H), 6.64 (d, J = 8.0 Hz, 2H), 4.29–4.20 (m, 3H), 3.92 (s, 2H), 1.34 (t, J = 6.4 Hz, 3H) ppm.

Ethyl *p*-tolylglycinate (3b)[5]

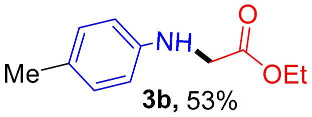

3b, 53%

Yield: 53%; Browish solid; mp = 56-58 °C; ^{1}H NMR (500 MHz, $CDCl_3$) δ: 7.01 (d, J = 7.6 Hz, 2H), 6.58 (d, J = 7.8 Hz, 2H), 4.26 (q, J = 6.8 Hz, 2H), 3.90 (s, 2H), 2.28 (s, 3H), 1.33 (t, J = 7.2 Hz, 3H) ppm.

Ethyl (4-methoxyphenyl)glycinate (3c)[5]

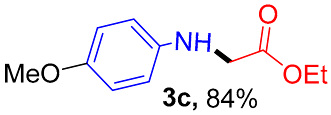

3c, 84%

Yield: 84%; Yellow semi-solid; mp = 43 °C; ^{1}H NMR (500 MHz, $CDCl_3$) δ: 6.78 (d, J = 8.2 Hz, 2H), 6.54 (d, J = 8.2 Hz, 2H), 4.22 (q, J = 6.8 Hz, 2H), 4.10 (bs, 1H), 3.86 (s, 2H), 3.75 (s, 3H), 1.31 (t, J = 7.0 Hz, 3H) ppm.

Ethyl (4-bromophenyl)glycinate (3d)[6]

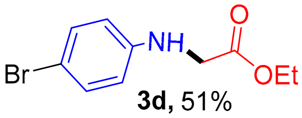

3d, 51%

Yield: 51%; Light reddish brown solid; mp = 95-98 °C; ^{1}H NMR (500 MHz, $CDCl_3$) δ: 7.30–7.23 (m, 2H), 6.51–6.46 (m, 2H), 4.11 (s, 1H), 4.27 (q, J = 7.0 Hz, 2H), 3.89 (s, 2H), 1.31 (t, J = 7.2 Hz, 3H) ppm.

Ethyl (4-cyanophenyl)glycinate (3e)[7]

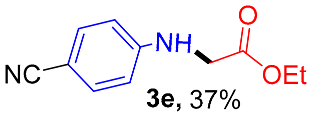

3e, 37%

Yield: 37%; Yellowish solid; ^{1}H NMR (500 MHz, $CDCl_3$) δ: δ: 7.48 (d, J = 7.8 Hz, 2H), 6.59 (d, J = 7.6 Hz, 2H), 4.08 (s, 1H), 4.28 (q, J = 7.1 Hz, 2H), 3.90 (s, 2H), 1.29 (t, J = 7.2 Hz, 3H) ppm.

Ethyl (4-(trifluoromethyl)phenyl)glycinate (3f)[8]

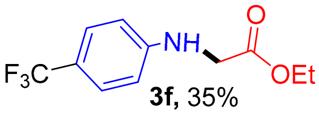

3f, 35%

Yield: 35%; Pale white solid; ^{1}H NMR (500 MHz, $CDCl_3$) δ: 7.40 (dd, J = 8.2, 1.2 Hz, 2H), 6.59 (dd, J = 8.4, 1.2 Hz, 2H), 4.60 (s, 1H), 4.20 (q, J = 7.0 Hz, 2H), 3.89 (s, 2H), 1.28 (t, J = 7.2 Hz, 3H) ppm.

Ethyl (4-nitrophenyl)glycinate (3g)[9]

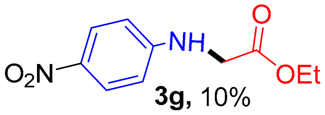

3g, 10%

Yield: 10%; Yellow solid; mp = 132-134 °C; ^{1}H NMR (500 MHz, $CDCl_3$) δ: 8.14 (d, J = 8.8 Hz, 2H), 6.60 (d, J = 8.8 Hz, 2H), 5.11 (bs, 1H), 4.30 (q, J =7.2 Hz, 2H), 3.40 (s, 2H), 1.33 (t, J = 7.0 Hz, 3H) ppm.

Ethyl naphthalen-1-ylglycinate (3h)[10]

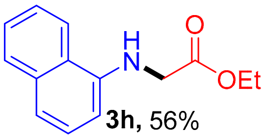

3h, 56%

Yield: 56%; White solid; ^{1}H NMR (500 MHz, $CDCl_3$) δ: δ: 7.45 (d, J = 7.2 Hz, 2H), 7.30 (m, 2H), 7.21 (m, 1H), 7.09 (m, 2H), 4.12 (s, 1H), 4.10 (q, J = 7.1 Hz, 2H), 3.89 (s, 2H), 1.28 (t, J = 7.2 Hz, 3H) ppm.

Ethyl benzoylglycinate (3i)[11]

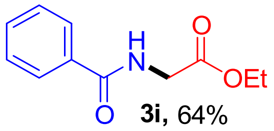
3i, 64%

Yield: 64%; Pale white solid; mp = 60 °C; ^{1}H NMR (500 MHz, $CDCl_3$) δ: 7.86 (d, J = 7.8 Hz, 2H), 7.50 (t, J = 7.0 Hz, 1H), 7.45 (dd, J = 8.0, 7.1 Hz, 2H), 6.80 (s, 1H), 4.30–4.22 (s, 4H), 1.34 (t, J = 7.2 Hz, 3H) ppm.

tert-Butyl 2-(phenylamino)acetate (3n)[12]

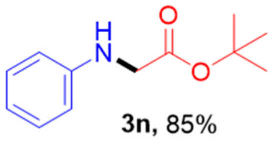
3n, 85%

Yield: 85%; Colorless oil; ^{1}H NMR (500 MHz, $CDCl_3$) δ: 7.18 (dd, J = 7.6 Hz, 2H), 6.73 (t, J = 7.6 Hz, 1H), 6.6 (d, J = 7.6 Hz, 2H), 3.24 (br, 1H), 3.79 (s, 2H), 1.48 (s, 9H) ppm.

Ethyl benzylglycinate (3o)[13]

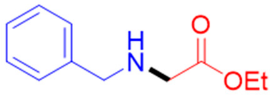
3o, 73%

Yield: 73%; Pale yellow oil; ^{1}H NMR (500 MHz, $CDCl_3$) δ: 7.46-7.17 (m, 5H), 4.05 (q, J = 7.1 Hz, 2H), 3.8 (s, 2H), 3.39 (s, 2H), 2.18 (bs, 1H), 1.26 (t, J = 7.1 Hz, 3H) ppm.

Ethyl (1-phenylethyl)glycinate (3p)[14]

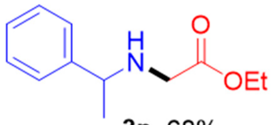
3p, 68%

Yield: 68%; Pale yellow oil; ^{1}H NMR (500 MHz, $CDCl_3$) δ: 7.32-7.24 (m, 5H), 4.15 (q, J = 7.2 Hz, 2H), 3.79 (q, J = 6.4, 2H), 2.25 (ABq, J_{AB} = 17.4 Hz, 2H), 1.87 (bs, 1H), 1.38 (d, J = 6.4 Hz, 3H), 1.24 (t, J = 7.2 Hz, 3H) ppm.

Ethyl 2-(piperidin-1-yl)acetate (3q)[15]

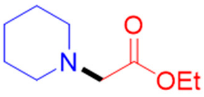

3q, 50%

Yield: 50%; Pale yellow oil; ^{1}H NMR (500 MHz, $CDCl_3$) δ: 4.18 (q, J = 7.2 Hz, 2H), 3.17 (s, 2H), 2.5 (m, 4H), 1.62 (m, 4H), 1.43 (m, 2H), 1.27 (t, J = 7.2 Hz, 3H) ppm.

Ethyl 2-morpholinoacetate (3r)[7]

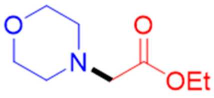

3r, 66%

Yield: 66%; Pale yellow oil; ^{1}H NMR (500 MHz, $CDCl_3$) δ: 4.10 (q, J = 7.2 Hz, 2H), 3.55 (m, 4H) 3.02 (s, 2H), 2.48 (m, 4H), 1.27 (t, J = 7.2 Hz, 3H) ppm.

Methyl 2-phenyl-2-(piperidin-1-yl)acetate (3s)[16]

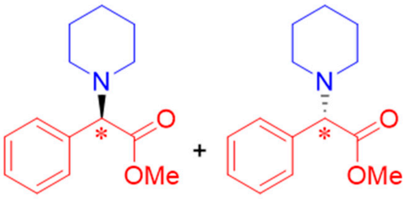

3s, 67%(1:1)

Yield: 67%; Pale yellow oil; ^{1}H NMR (500 MHz, $CDCl_3$) δ: 7.25-7.35 (m, 3H), 7.39-7.44 (m, 2H), 3.97 (s, 1H), 3.66 (s, 3H), 2.28-2.45 (m, 4H), 1.52-1.64 (m, 4H), 1.37-1.47 (m, 2H) ppm.

Methyl 2-morpholino-2-phenylacetate (3t)[16]

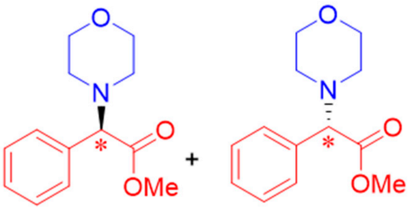

3t, 78%(1:1)

Yield: 78%; Pale yellow oil; ^{1}H NMR (500 MHz, $CDCl_3$) δ: 7.42-7.46 (m, 2H), 7.29-7.38 (m, 3H), 3.98 (s, 1H), 3.73 (t, J = 5.2 Hz 4H), 3.69 (s, 3H), 2.45 (t, J = 4 Hz 4H) ppm.

Methyl 2-phenyl-2-(phenylamino)acetate (3u)[17]

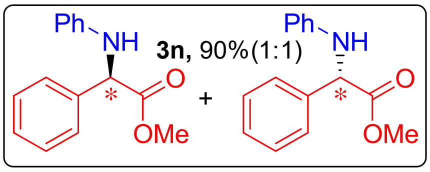

Yield: 90%; Yellowish solid; ^{1}H NMR (500 MHz, $CDCl_3$) δ: 7.52 (d, J = 7.0 Hz, 2H), 7.40–7.36 (m, 3H), 7.15 (t, J = 7.6 Hz, 2H), 6.69 (t, J = 7.6 Hz, 1H), 6.60 (d, J = 7.6 Hz, 2H), 5.06 (d, J = 6.0 Hz, 1H), 4.98 (s, 1H), 4.31–4.20 (s, 2H), 1.25 (t, J = 7.1 Hz, 3H) ppm.

Methyl 2-(4-fluorophenyl)-2-(phenylamino)acetate (3v)

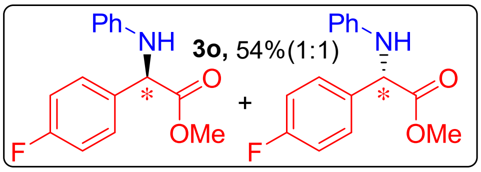

Yield: 56%; Yellow sticky solid; IR (neat) 3218, 3051, 2967, 2954, 1684, 1626, 1591, 1447, 1368, 1218, 1030, 785 cm^{-1}; ^{1}H NMR (500 MHz, $CDCl_3$) δ:7.53–7.47 (m, 2H), 7.15 (t, J = 8.0 Hz, 2H), 7.06 (t, J = 8.5 Hz, 2H), 6.73 (t, J = 7.5 Hz, 2H), 6.51 (d, J = 3.0 Hz, 1H), 5.07 (d, J = 5.5 Hz, 1H), 4.98 (bs, 1H), 3.75 (s, 3H) ppm; ^{13}C NMR (125 MHz, $CDCl_3$) δ:172.1, 163.7 ($^1J_{C-F}$ = 245.6 Hz), 145.7, 133.4 ($^4J_{C-F}$ = 3.1 Hz), 129.3, 129.0 7 ($^3J_{C-F}$ = 8.3 Hz), 118.3, 115.9 ($^2J_{C-F}$ = 21.5 Hz), 113.4, 60.0, 52.9 ppm; ^{19}F NMR (500 MHz, $CDCl_3$) δ: 113.86 ppm; HRMS (ESI): calcd for $C_{15}H_{14}FNaNO_2$: 282.0901 [M+H$^+$]; found: 282.0948.

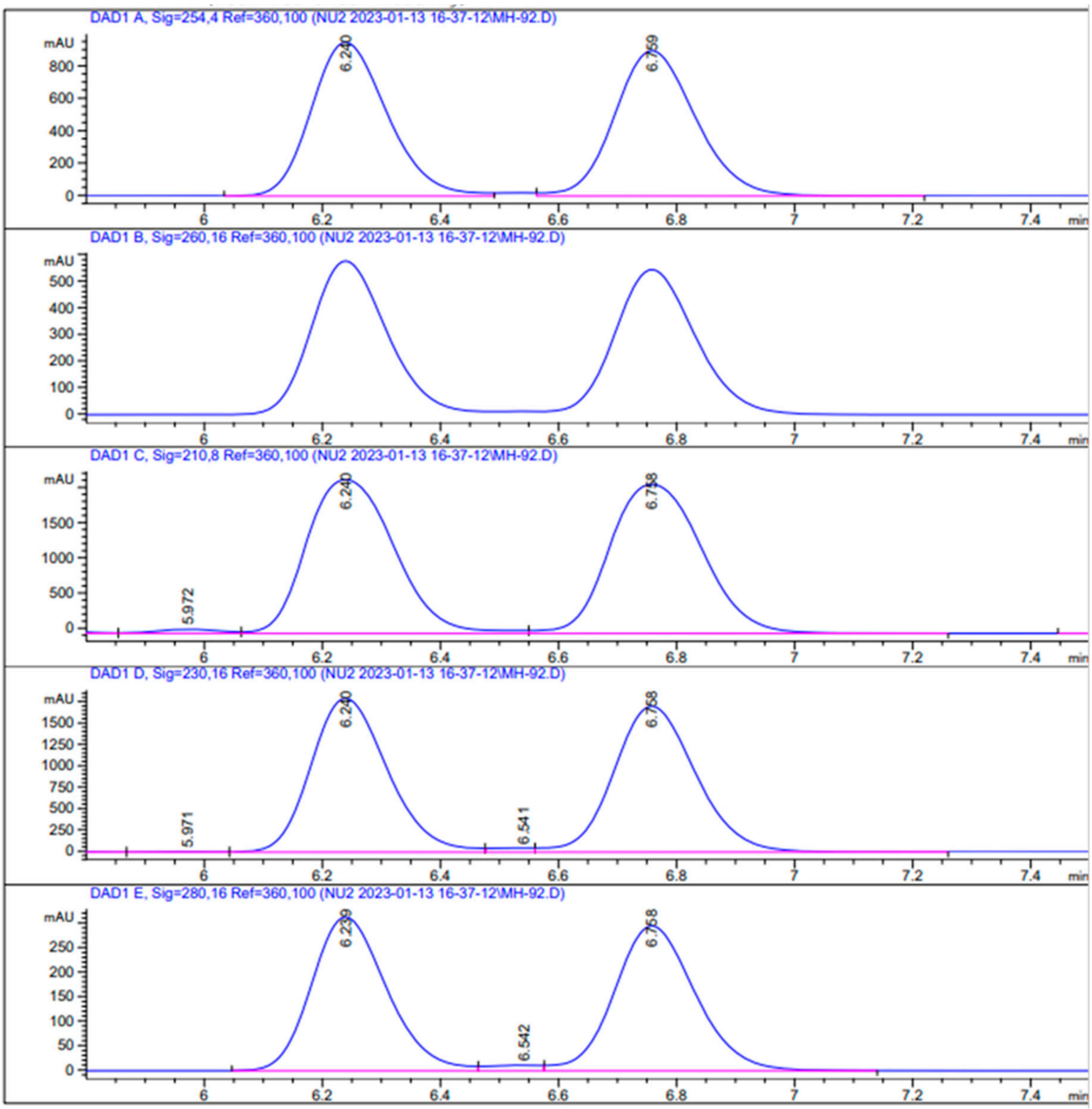

Signal 1: DAD1 A, Sig=254,4 Ref=360,100

Peak #	RetTime [min]	Type	Width [min]	Area [mAU*s]	Height [mAU]	Area %
1	6.240	VV	0.1386	8516.69531	950.15955	49.8175
2	6.759	VB	0.1495	8579.08691	897.73230	50.1825
Totals :				1.70958e4	1847.89185	

Figure S3. HPLC spectra of compound **3v** and integration data.

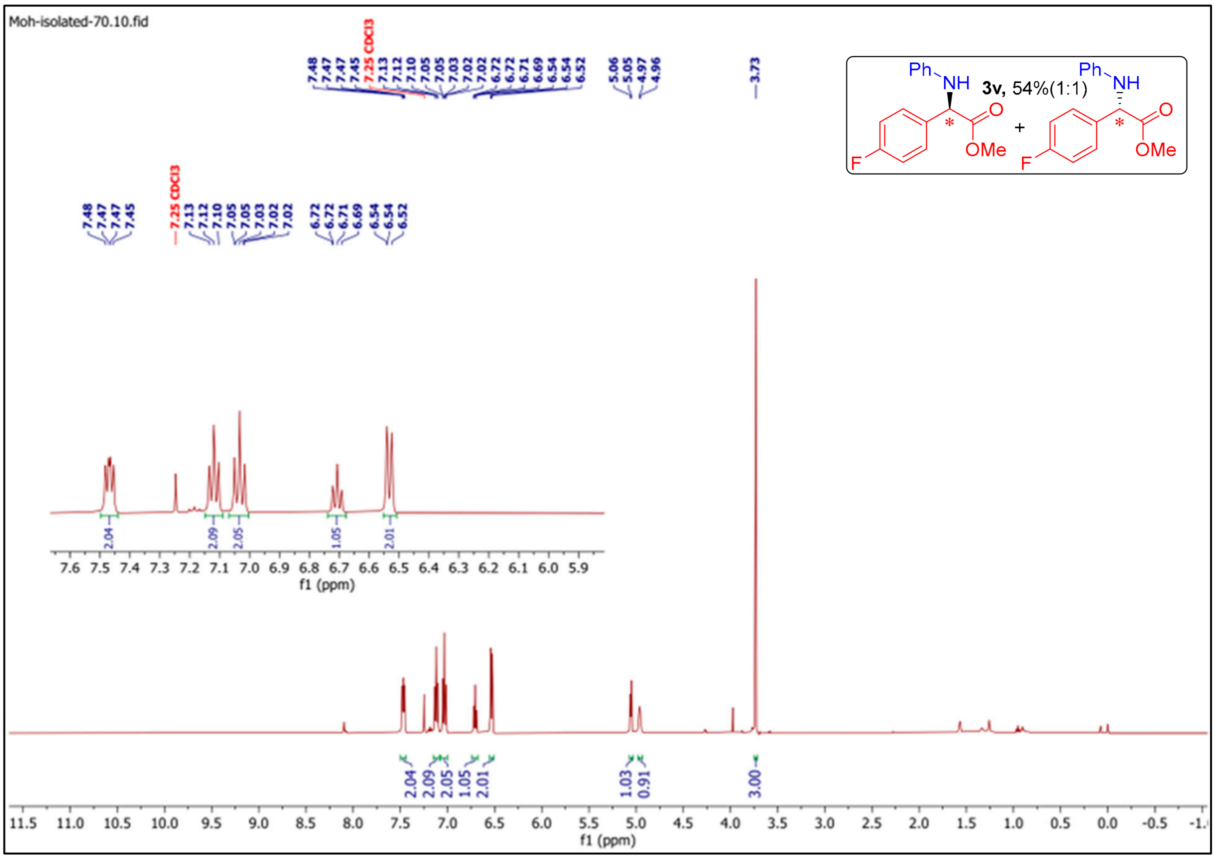

Figure S4. ^{1}H NMR spectra of compound **3v (CDCl$_3$, 500 MHz)**

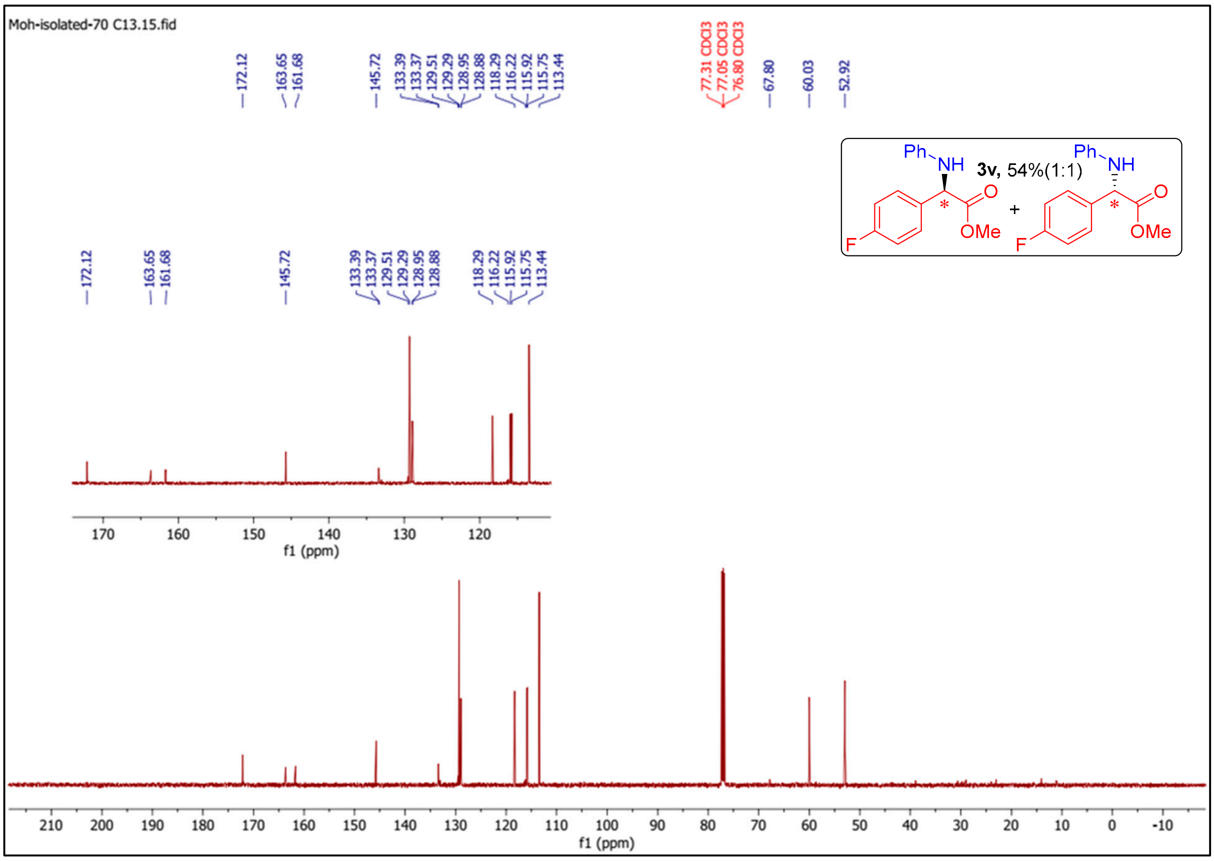

Figure S5. 13C NMR spectra of compound **3v (CDCl₃, 125 MHz)**

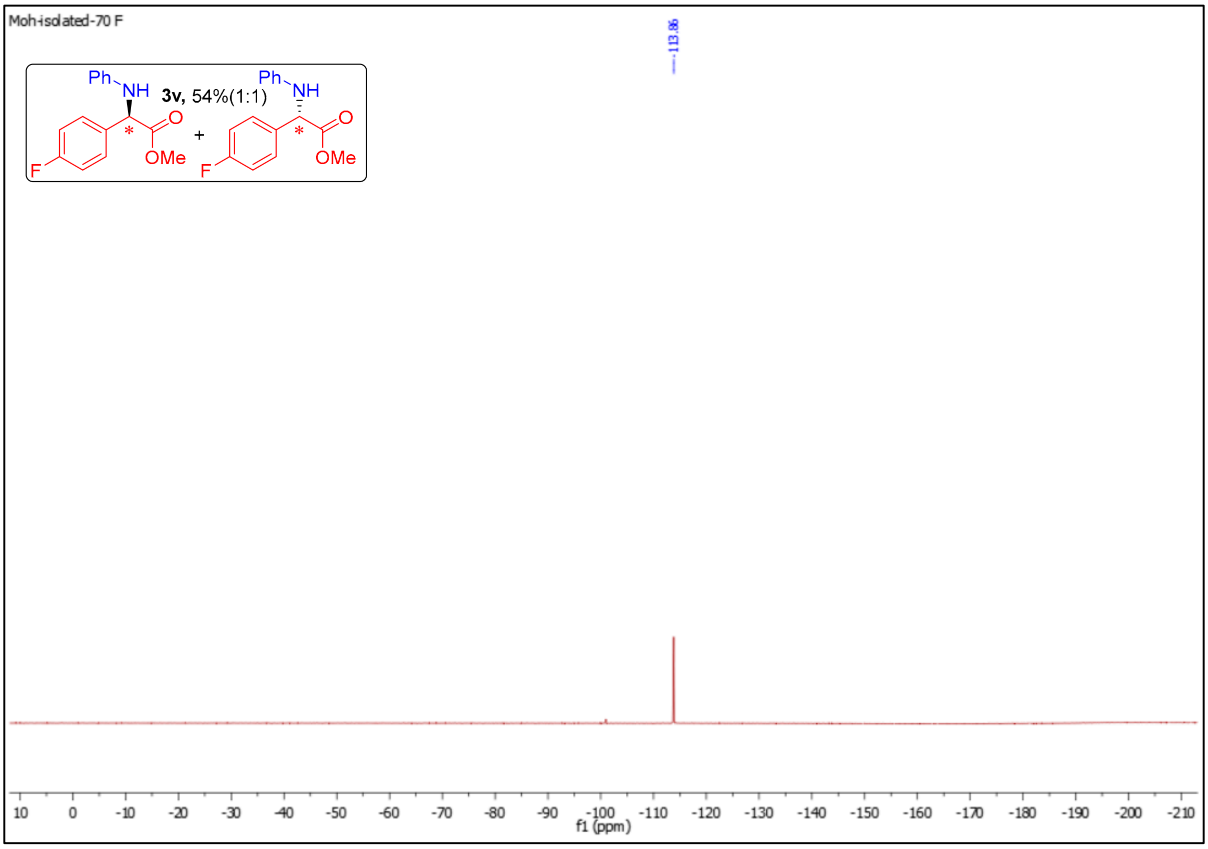

Figure S6. ^{19}F NMR spectra of compound **3v (CDCl$_3$, 40 MHz)**

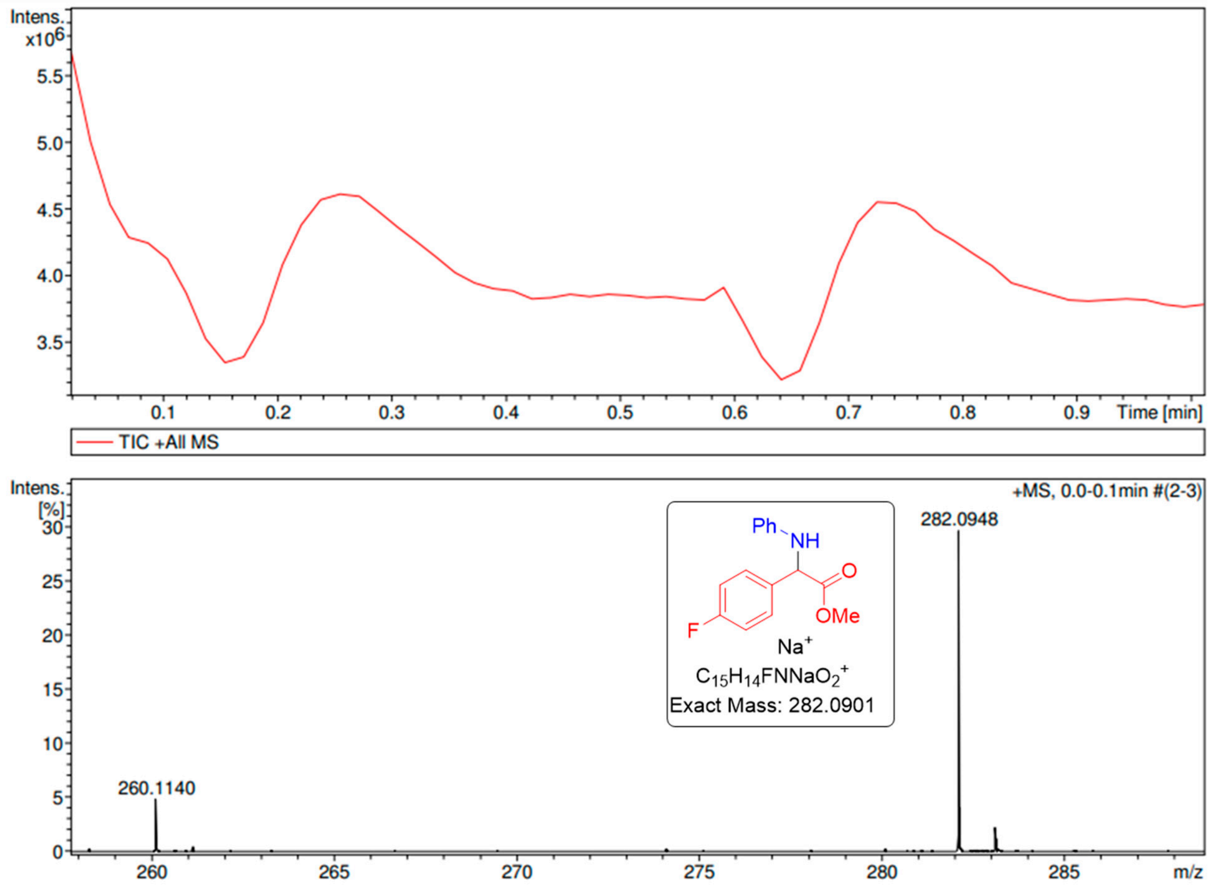

Figure S7. HRMS data for the compound **3o**

Cu^{2+}(PQM) catalyst N–H insertion reaction kinetics plot for compound 3a

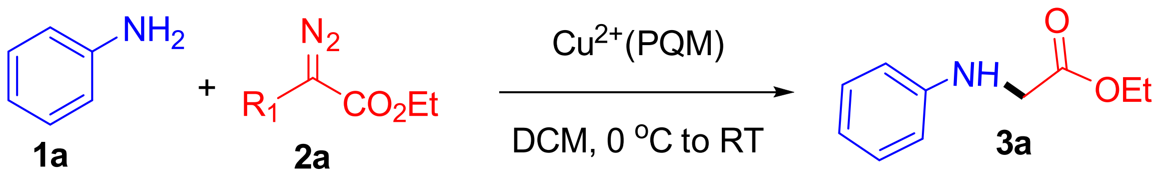

A 1.0 mL NMR tube was charged with 0.5 mmol of anilines (**1a**), 1.0 mmol of ethyl diazo acetate (**2a**), 0.05 mmol of mesitylene (used as an internal standard) and 0.05 mmol of Cu^{2+}(PQM). This mixture was dissolved in 0.5 mL of deuterated dichloromethane (DCM-d_2), and the reaction was started at 0 °C to ambient temperature stir for approximately five hours under an N_2 atmosphere. The reaction progress was monitored at 10-minute intervals using a 500 MHz NMR instrument.

Time (min)	30	60	90	120	150	180	210	240	270	300
Yields (%)	12	21	38	47	59	64	70	73	73	74

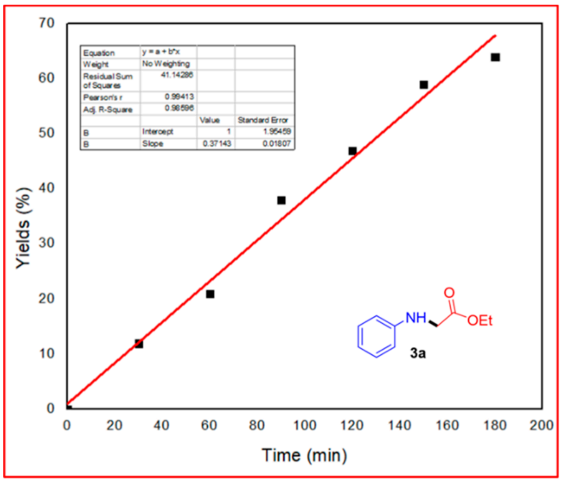

Figure S8. N–H insertion reaction kinetics plot for compound **3a**.

Cu^{2+}(PQM) catalyst N–H insertion reaction kinetics plot for compound 3b

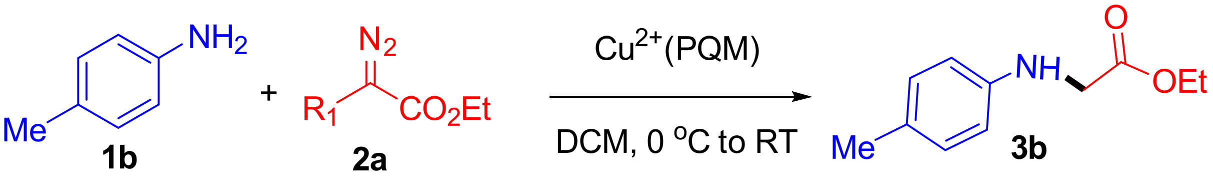

A 1.0 mL NMR tube was charged with 0.5 mmol of anilines (**1b**), 1.0 mmol of ethyl diazo acetate (**2a**), 0.05 mmol of mesitylene (used as an internal standard) and 0.05 mmol of Cu^{2+}(PQM). This mixture was dissolved in 0.5 mL of deuterated dichloromethane (DCM-d_2), and the reaction was started at 0 °C to ambient temperature stir for approximately five hours under an N_2 atmosphere. The reaction progress was monitored at 10-minute intervals using a 500 MHz NMR instrument.

Time (min)	30	60	90	120	150	180	210	240	270	300
Yields (%)	9	15	23	31	38	43	47	53	53	54

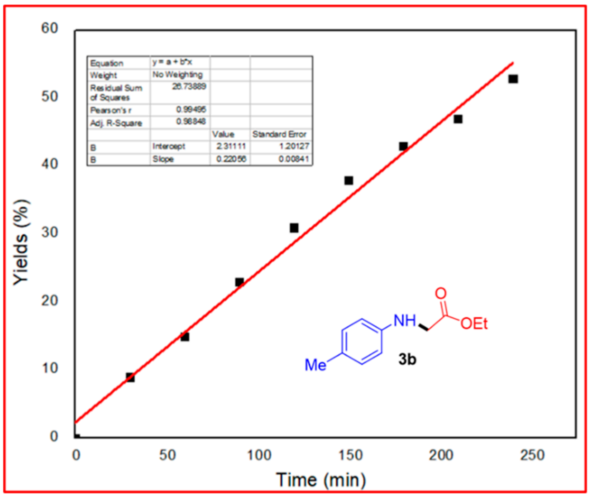

Figure S9. N–H insertion reaction kinetics plot for compound **3b**.

Cu^{2+}(PQM) catalyst N–H insertion reaction kinetics plot for compound 3c

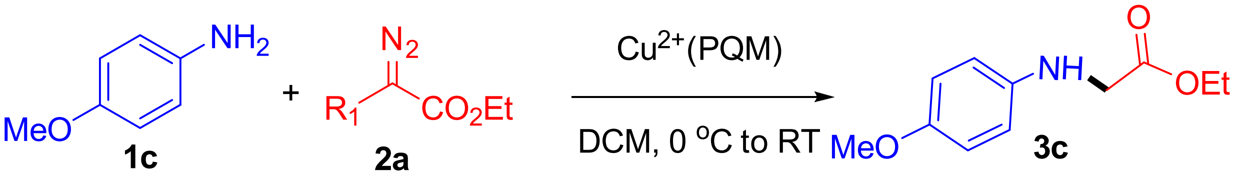

A 1.0 mL NMR tube was charged with 0.5 mmol of anilines (**1c**), 1.0 mmol of ethyl diazo acetate (**2a**), 0.05 mmol of mesitylene (used as an internal standard) and 0.05 mmol of Cu^{2+}(PQM). This mixture was dissolved in 0.5 mL of deuterated dichloromethane (DCM-d_2), and the reaction was started at 0 °C to ambient temperature stir for approximately five hours under an N_2 atmosphere. The reaction progress was monitored at 10-minute intervals using a 500 MHz NMR instrument.

Time (min)	30	60	90	120	150	180	210	240	270	300
Yields (%)	15	31	45	59	65	71	79	83	83	83

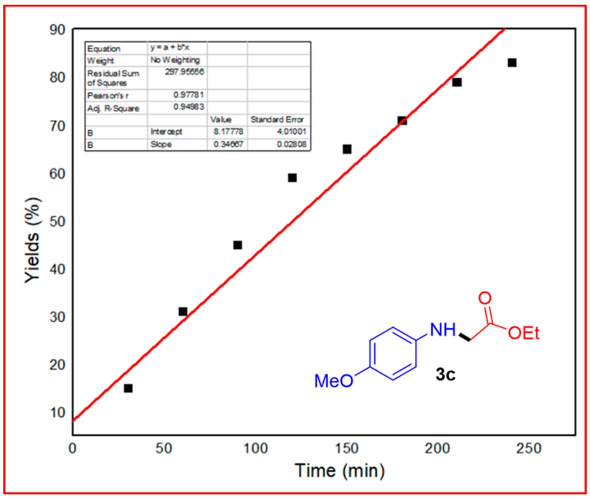

Figure S10. N–H insertion reaction kinetics plot for compound **3c**.

Cu^{2+}(PQM) catalyst N–H insertion reaction kinetics plot for compound 3d

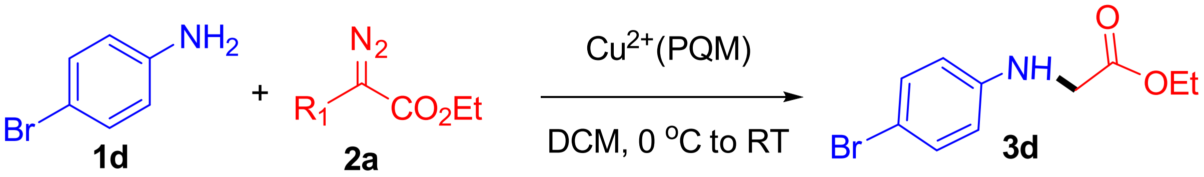

A 1.0 mL NMR tube was charged with 0.5 mmol of anilines (**1d**), 1.0 mmol of ethyl diazo acetate (**2a**), 0.05 mmol of mesitylene (used as an internal standard) and 0.05 mmol of Cu^{2+}(PQM). This mixture was dissolved in 0.5 mL of deuterated dichloromethane (DCM-d_2), and the reaction was started at 0 °C to ambient temperature stir for approximately five hours under an N_2 atmosphere. The reaction progress was monitored at 10-minute intervals using a 500 MHz NMR instrument.

Time (min)	30	60	90	120	150	180	210	240	270	300
Yields (%)	11	18	23	31	38	45	49	51	51	51

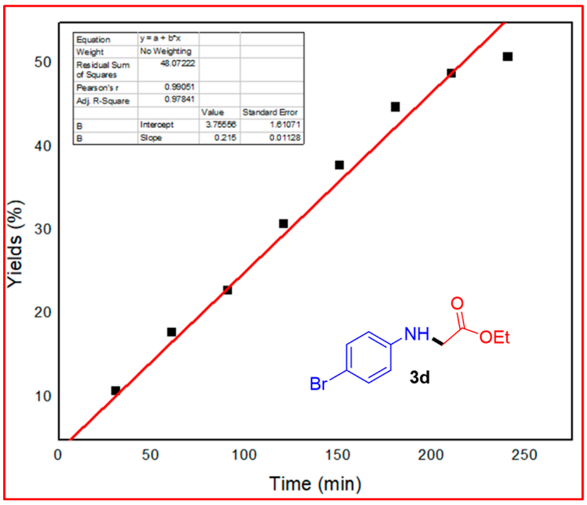

Figure S11. N–H insertion reaction kinetics plot for compound **3d**.

Cu^{2+}(PQM) catalyst N–H insertion reaction kinetics plot for compound 3e

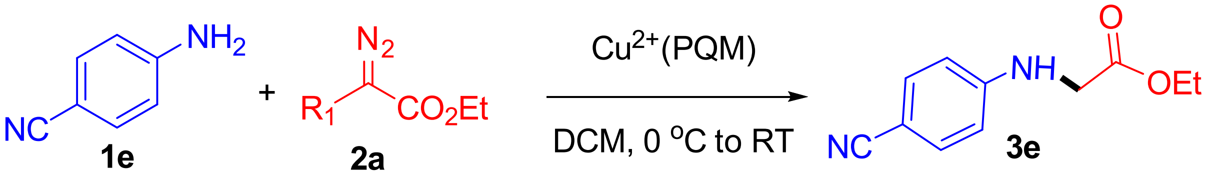

A 1.0 mL NMR tube was charged with 0.5 mmol of anilines (**1e**), 1.0 mmol of ethyl diazo acetate (**2a**), 0.05 mmol of mesitylene (used as an internal standard) and 0.05 mmol of Cu^{2+}(PQM). This mixture was dissolved in 0.5 mL of deuterated dichloromethane (DCM-d_2), and the reaction was started at 0 °C to ambient temperature stir for approximately five hours under an N_2 atmosphere. The reaction progress was monitored at 10-minute intervals using a 500 MHz NMR instrument.

Time (min)	30	60	90	120	150	180	210	240	270	300
Yields (%)	4	9	14	21	27	30	34	37	38	39

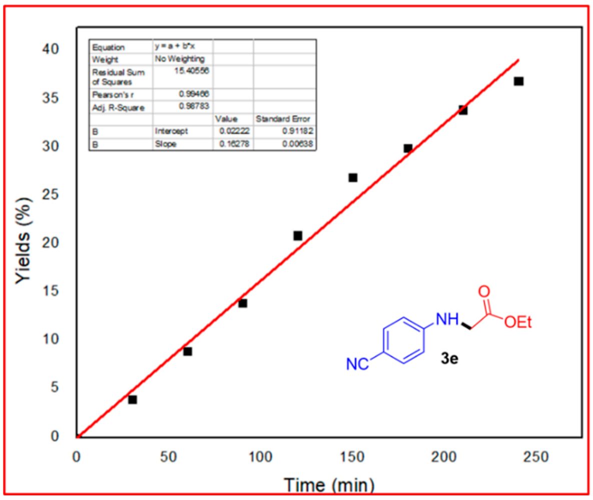

Figure S12. N–H insertion reaction kinetics plot for compound **3e**.

Cu^{2+}(PQM) catalyst N–H insertion reaction kinetics plot for compound 3f

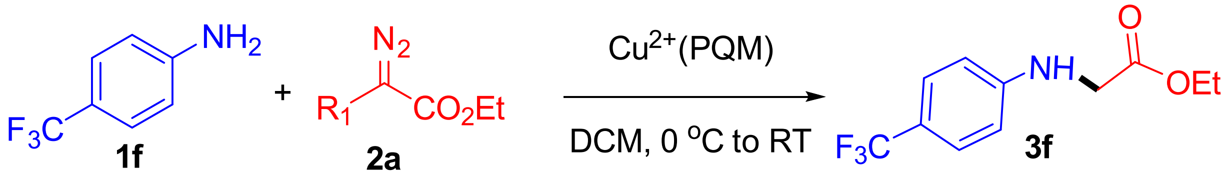

A 1.0 mL NMR tube was charged with 0.5 mmol of anilines (**1f**), 1.0 mmol of ethyl diazo acetate (**2a**), 0.05 mmol of mesitylene (used as an internal standard) and 0.05 mmol of Cu^{2+}(PQM). This mixture was dissolved in 0.5 mL of deuterated dichloromethane (DCM-d_2), and the reaction was started at 0 °C to ambient temperature stir for approximately five hours under an N_2 atmosphere. The reaction progress was monitored at 10-minute intervals using a 500 MHz NMR instrument.

Time (mints)	30	60	90	120	150	180	210	240	270	300
Yields (%)	4	9	14	21	27	30	34	37	38	39

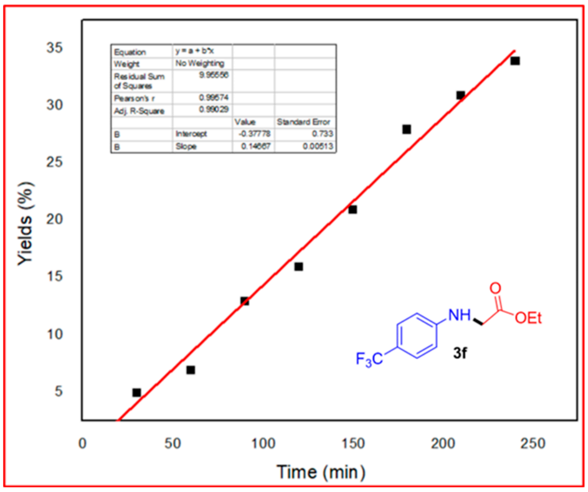

Figure S13. N–H insertion reaction kinetics plot for compound **3f**.

Cu^{2+}(PQM) catalyst N–H insertion reaction kinetics plot for compound 3g

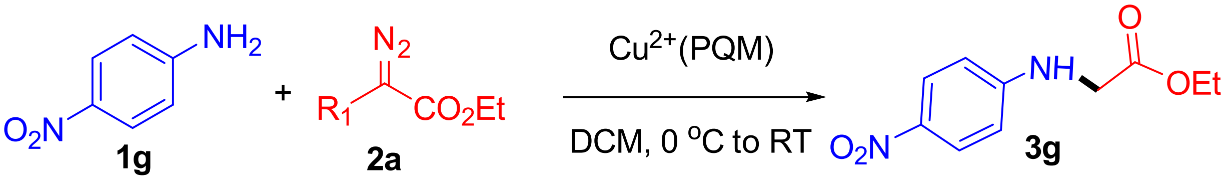

A 1.0 mL NMR tube was charged with 0.5 mmol of anilines (**1g**), 1.0 mmol of ethyl diazo acetate (**2a**), 0.05 mmol of mesitylene (used as an internal standard) and 0.05 mmol of Cu^{2+}(PQM). This mixture was dissolved in 0.5 mL of deuterated dichloromethane (DCM-d_2), and the reaction was started at 0 °C to ambient temperature stir for approximately five hours under an N_2 atmosphere. The reaction progress was monitored at 10-minute intervals using a 500 MHz NMR instrument.

Time (mints)	30	60	90	120	150	180	210	240	270	300
Yields (%)	0	2	3	5	7	9	10	10	11	11

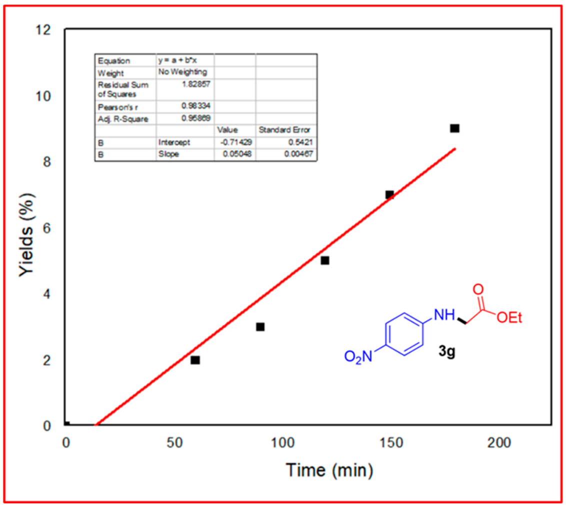

Figure S14. N–H insertion reaction kinetics plot for compound **3g**.

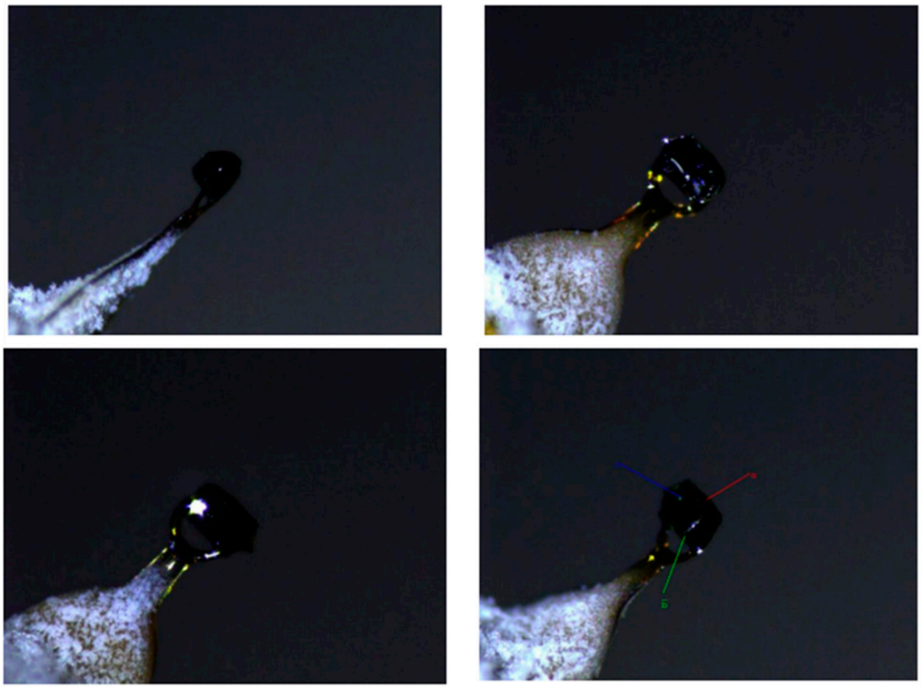

Figure S15: Cu^{2+}(PQM) complex crystal view on mounted on a cryoLoop.

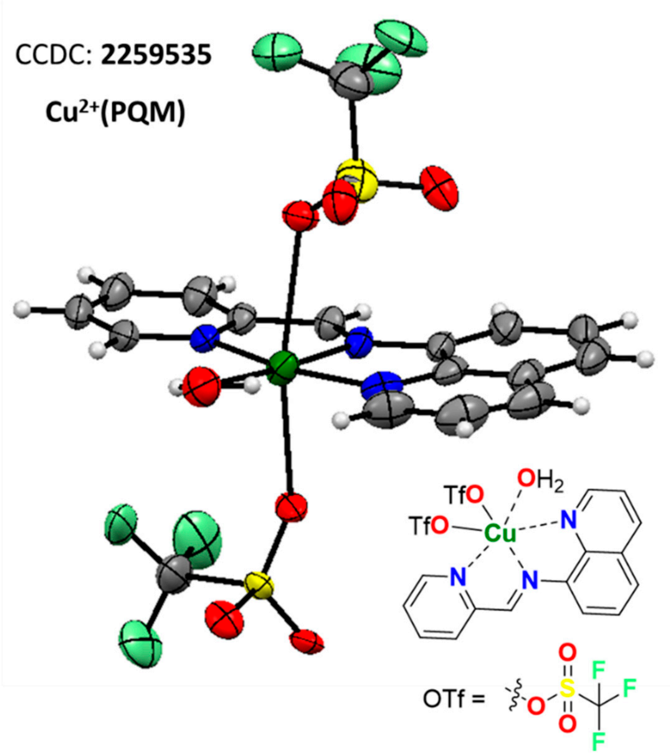

Figure S16. Crystallographic data, structure of Cu^{2+}(PQM) complex, and related report

Table S1: Sample and crystal data for **Cu^{2+}(PQM)** complex.

**Identification code**	D_JE_014	
**Chemical formula**	$C_{17}H_{19}CuF_6N_3O_{10}S_2$	
**Formula weight**	667.01 g/mol	
**Temperature**	120(2) K	
**Wavelength**	1.54178 Å	
**Crystal size**	(0.168 x 0.267 x 0.271) mm^3	
**Crystal system**	monoclinic	
**Space group**	C 1 2/c 1	
**Unit cell dimensions**	a = 24.0665(11) Å	α = 90°
	b = 11.8732(5) Å	β = 99.940(2)°
	c = 17.8506(8) Å	γ = 90°
**Volume**	5024.2(4) $Å^3$	
**Z**	8	
**Density (calculated)**	1.764 g/cm^3	
**Absorption coefficient**	3.742 mm^{-1}	
**F(000)**	2696	

Table S2. Data collection and structure refinement for Cu^{2+}(PQM) complex.

**Theta range for data collection**	3.73 to 74.66°
**Index ranges**	-30<=h<=30, -14<=k<=14, -22<=l<=22
**Reflections collected**	48142
**Independent reflections**	5136 [R(int) = 0.0327]
**Coverage of independent reflections**	99.7%
**Absorption correction**	Multi-Scan
**Max. and min. transmission**	0.5720 and 0.4300
**Refinement method**	Full-matrix least-squares on F^2
**Refinement program**	SHELXL-2018/3 (Sheldrick, 2018)

**Function minimized**	$\Sigma\, w(F_o^2 - F_c^2)^2$
**Data / restraints / parameters**	5136 / 585 / 493
**Goodness-of-fit on F²**	1.048
**Final R indices**	4785 data; I>2σ(I) R1 = 0.0803, wR2 = 0.2308
	all data R1 = 0.0837, wR2 = 0.2349
**Weighting scheme**	$w=1/[\sigma^2(F_o^2)+(0.1416P)^2+35.7616P]$ where $P=(F_o^2+2F_c^2)/3$
**Extinction coefficient**	0.0004(1)
**Largest diff. peak and hole**	2.426 and -1.014 eÅ^{-3}
**R.M.S. deviation from mean**	0.149 eÅ^{-3}

References:

1. Lions, F.; Martin, K. V Tridentate Chelate Compounds. I. *J. Am. Chem. Soc.* **1957**, *79*, 2733–2738, doi:10.1021/ja01568a018.

2. Kinunda, G.; Jaganyi, D. Understanding the Electronic and π-Conjugation Roles of Quinoline on Ligand Substitution Reactions of Platinum(II) Complexes. *Transit. Met. Chem.* **2014**, *39*, 451–459, doi:10.1007/s11243-014-9819-8.

3. Sorouraddin, M.-H.; Rashidi, M.-R.; Shabani, B.; Ghorbani-Kalhor, E. Spectrofluorimetric Determination of Cu2+ Using New Fluorogenic Reagent. *Chinese J. Chem.* **2005**, *23*, 160–165, doi:https://doi.org/10.1002/cjoc.200590160.

4. D. Quach, T.; A. Batey, R. Ligand- and Base-Free Copper(II)-Catalyzed C−N Bond Formation: Cross-Coupling Reactions of Organoboron Compounds with Aliphatic Amines and Anilines. *Org. Lett.* **2003**, *5*, 4397–4400, doi:10.1021/ol035681s.

5. Sreenilayam, G.; Fasan, R. Myoglobin-Catalyzed Intermolecular Carbene N--H Insertion with Arylamine Substrates. *Chem Comm* **2015**, *51*, 1532–1534.

6. Ouyang, L.; Li, J.; Zheng, J.; Huang, J.; Qi, C.; Wu, W.; Jiang, H. Access to α-Amino Acid Esters through Palladium-Catalyzed Oxidative Amination of Vinyl Ethers with Hydrogen Peroxide as the Oxidant and Oxygen Source. *Angew. Chemie Int. Ed.* **2017**, *56*, 15926–15930, doi:https://doi.org/10.1002/anie.201709285.

7. Aviv, I.; Gross, Z. Iron(III) Corroles and Porphyrins as Superior Catalysts for the Reactions of Diazoacetates with Nitrogen- or Sulfur-Containing Nucleophilic Substrates: Synthetic Uses and Mechanistic Insights. *Chem. – A Eur. J.* **2008**, *14*, 3995–4005, doi:https://doi.org/10.1002/chem.200701885.

8. Rohlmann, R.; Stopka, T.; Richter, H.; García Mancheño, O. Iron-Catalyzed Oxidative Tandem Reactions with TEMPO Oxoammonium Salts: Synthesis of Dihydroquinazolines and Quinolines. *J. Org. Chem.* **2013**, *78*, 6050–6064, doi:10.1021/jo4007199.

9. Wolf, M.W.; Vargas, D.A.; Lehnert, N. Engineering of RuMb: Toward a Green Catalyst for Carbene Insertion Reactions. *Inorg. Chem.* **2017**, *56*, 5623–5635, doi:10.1021/acs.inorgchem.6b03148.

10. Abu-Elfotoh, A.-M. NH Insertion Reactions Catalyzed by Reusable Water-Soluble Ruthenium(II)-Hm-Phenyloxazoline Complex. *Tetrahedron Lett.* **2017**, *58*, 4750–4754, doi:https://doi.org/10.1016/j.tetlet.2017.10.062.

11. Ueda, T.; Konishi, H.; Manabe, K. Trichlorophenyl Formate: Highly Reactive and Easily Accessible Crystalline CO Surrogate for Palladium-Catalyzed Carbonylation of Aryl/Alkenyl Halides and Triflates. *Org. Lett.* **2012**, *14*, 5370–5373, doi:10.1021/ol302593z.

12. Hattori, G.; Sakata, K.; Matsuzawa, H.; Tanabe, Y.; Miyake, Y.; Nishibayashi, Y. Copper-Catalyzed Enantioselective Propargylic Amination of Propargylic Esters with Amines: Copper−Allenylidene Complexes as Key Intermediates. *J. Am. Chem. Soc.* **2010**, *132*, 10592–10608, doi:10.1021/ja1047494.

13. Ugarriza, I.; Uria, U.; Carrillo, L.; Vicario, J.L.; Reyes, E. Base-Promoted C→N Acyl Rearrangement: An Unconventional Approach to α-Amino Acid Derivatives. *Chem. – A Eur. J.* **2014**, *20*, 11650–11654, doi:https://doi.org/10.1002/chem.201402514.

14. Steck Gopeekrishnan; Fasan Rudi, V.S. Selective Functionalization of Aliphatic Amines via Myoglobin-Catalyzed Carbene N–H Insertion. *Synlett* **2020**, *31*, 224–229, doi:10.1055/s-0039-1690007.

15. Baumann, L.K.; Mbuvi, H.M.; Du, G.; Woo, L.K. Iron Porphyrin Catalyzed N−H Insertion Reactions with Ethyl Diazoacetate. *Organometallics* **2007**, *26*, 3995–4002, doi:10.1021/om0610997.

16. Miura, T.; Morimoto, M.; Murakami, M. Copper-Catalyzed Amination of Silyl Ketene Acetals with N-Chloroamines. *Org. Lett.* **2012**, *14*, 5214–5217, doi:10.1021/ol302331k.

17. Deng, Q.-H.; Xu, H.-W.; Yuen, A.W.-H.; Xu, Z.-J.; Che, C.-M. Ruthenium-Catalyzed One-Pot Carbenoid N−H Insertion Reactions and Diastereoselective Synthesis of Prolines. *Org. Lett.* **2008**, *10*, 1529–1532, doi:10.1021/ol800087p.
